# Population Structure, Demographic History and Maladaptation Risk in Ponderosa Pine

**DOI:** 10.3390/plants15213280

**Published:** 2026-10-24

**Authors:** Sean M. Collins, Anna Lincoln, Amanda R. De La Torre

**Affiliations:** 1 School of Forestry, Northern Arizona University, Flagstaff, AZ 86011, USA; smc2266@nau.edu; 2 Bureau of Land Management, Grand Junction, CO 81501, USA

**Keywords:** exome capture, *Pinus ponderosa*, demographic history, genome–environment association, genomic offset

## Abstract

The influence of demography and selection over a species’ evolutionary time can influence responses to current and future stressors. We used range-wide datasets in ponderosa pine (*Pinus ponderosa*) to understand the influence of demography and selection on current population structure. Samples were sequenced with 55,000 capture-seq probes, producing 1.2–1.5 million SNPs (~400 Mbp raw data per sample). We examined current population structure, changes in effective population size, evidence for selection, genome–environment associations and future maladaptation risk using genomic offset. Our results show support for four genetic clusters within the species, numerous bottleneck events that mirror glacial advances and past droughts, signatures of purifying and balancing selection, important environmental variables for climate adaptation, and many geographic areas with high amounts of genomic offset. Genetic differentiation was associated with two processes: (1) bottleneck-driven allele loss and reduced connectivity; and (2) spatially heterogeneous selection linked to moisture gradients. Many populations are predicted to be at risk of future maladaptation, and these populations coincide with geographic areas showing increased mortality events and decreased regeneration in previous studies. Large shifts in allele frequencies may be required for populations to track projected climates.

## 1. Introduction

Natural selection and gene flow play important roles in shaping species’ population structure and adaptive potential. Demographic events, selection, and gene flow often have confounding effects on genetic differentiation [1,2]. Environmental heterogeneity can lead to the selection of specific variants in different environments, leading populations to become differentiated due to adaptation to their local environment [3,4,5,6]. High gene flow can limit this differentiation across heterogeneous environments due to gene swamping, where advantageous alleles are less likely to become fixed [7,8]. However, in the presence of strong selection, advantageous alleles could be maintained across heterogeneous environments under high gene flow [9]. Evolutionary theory and empirical work suggest that a species can develop local adaptation through a variety of pathways and maintain advantageous alleles in several ways. Therefore, untangling the interplay between selection and demographic history can help understand how genetic variation and local adaptation are maintained in a species, and allow inferences to be made about species’ responses to new biotic and abiotic threats.

Ponderosa pine (*Pinus ponderosa*) is a long-generation, slow-growing, outcrossing, wind-dispersed conifer that is distributed from West Texas to British Columbia and exists across extremely wide ecological amplitudes, e.g., populations can exist in areas receiving 400 to 1100 mm mean of annual precipitation [10,11]. Due to its wide distribution, ponderosa pine provides biodiversity and ecosystem services, such as supporting rare or endangered species [12,13] and maintaining a sustainable water yield [14,15]. While readily abundant in many areas of North America, ponderosa pine has a very unclear population structure, with previous studies suggesting between two and nine intraspecific varieties using SSRs [16,17,18,19,20]. Two varieties are widely accepted, the coastal variety distributed from California to Idaho (variety *Ponderosa*) and the interior variety distributed from West Texas to Montana (variety *Scopulorum*). Some studies have split the coastal variety into two, with *Critchfieldiana* in California and the Pacific northwest and *Ponderosa* in the inland northwest [16,17,18,19,20]. Similarly, the interior variety has been split into three varieties: *Brachyptera* in the southwest, *Scopulorum* in Montana, Wyoming, northern Utah and northern Colorado, and *Readiana* in the Great Plains [16,17,18,19,20].

Range-wide genome-wide assessments have not been conducted for ponderosa pine due to the sequencing and computational challenges of working with very large datasets required to understand large, complex conifer genomes. The genome assembly is not currently available for ponderosa pine, but the species is diploid with an estimated genome size of 20–22 Gbp. Limited work has examined evolutionary forces shaping population structure and the genomic basis of climate adaptation in ponderosa pine [21,22,23,24,25]. Furthermore, increased mortality and decreased regeneration across the species range have been reported in the past decades most likely due to changing climate regimes [26,27,28]. To address these knowledge gaps, we aimed to (1) elucidate the population structure across the natural range of Ponderosa pine, (2) assess the role of demographic history in shaping current population structure, (3) identify environmental factors acting as selection pressures, and (4) identify populations at high risk of future maladaptation.

## 2. Results

### 2.1. Population Structure

**Figure 1:**
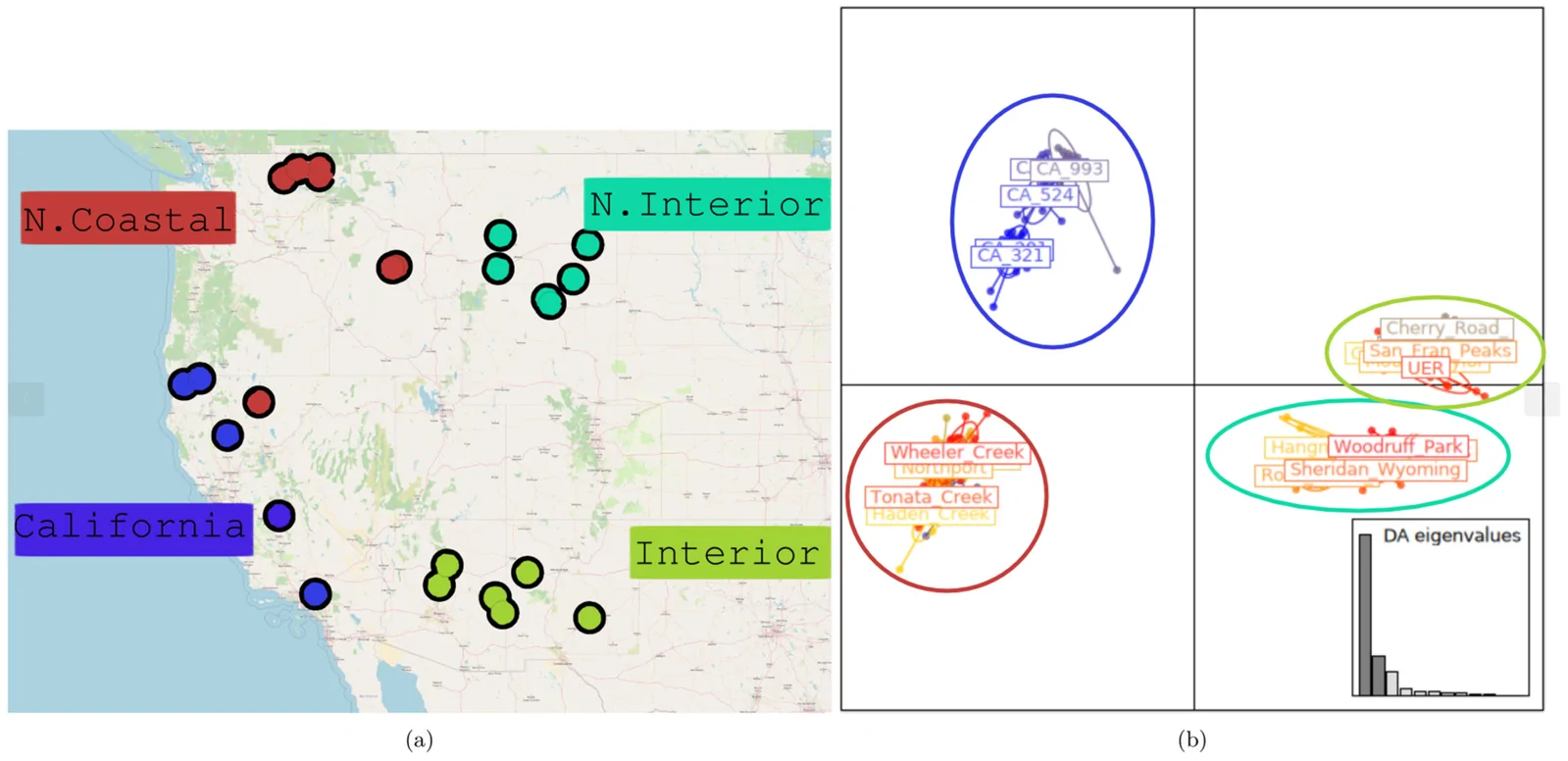
Population structure of Ponderosa pine. (**a**) Seed sources from dataset two are colored by genetic cluster from the DAPC results. (**b**) DAPC with seed source as population label. Top left cluster is the California cluster consisting of most of the California seed sources, bottom left cluster is the northern coastal cluster consisting of eastern Washington, Idaho and Northeastern California seed sources, top right cluster is the interior cluster consisting of Arizona and New Mexico seed sources, and bottom right is the northern interior cluster consisting of Montana and Wyoming seed sources.

Our results show support for four distinct genetic clusters (Figure 1, Supplementary Materials Figure S1). DAPC results show a strong split within the coastal portion of the US, with populations in the Northwest being distinct from most of California (Figure 1, Supplementary Materials Figure S1). Two clusters in the coastal portion differentiate in northern California or southern Oregon (Figure 1); thereby suggesting a California cluster and a northern coastal cluster. Our results also show differentiation in the interior west (Figure 1, Supplementary Materials Figure S1). DAPC results indicate that individuals in Montana and Wyoming are unique relative to the rest of the interior portion of the range; suggesting an interior cluster in the southwest and a northern interior cluster in Montana and Wyoming. Pairwise Fst supports observed patterns in population structure found in the DAPC (Supplementary Materials Figure S2, Table S1). Pairwise Fst also shows potential substructuring within genetic clusters, suggesting that some populations exchange more gene flow with certain areas within their clusters than with others (Supplementary Materials Figure S2, Table S1). These substructure clusters tend to mirror geographic distance, that is areas closer together tend to aggregate in the same split of the tree (Supplementary Materials Figure S2, Table S1). K-means clustering shows that the BIC continually decreases with added Ks (Supplementary Materials Table S2). The analysis of molecular variance (AMOVA) for dataset two shows that the mean square differentiation (MSD) for population was 0.164 and the MSD for the error term was 0.002.

### 2.2. Population Genomic Metrics

**Figure 2:**
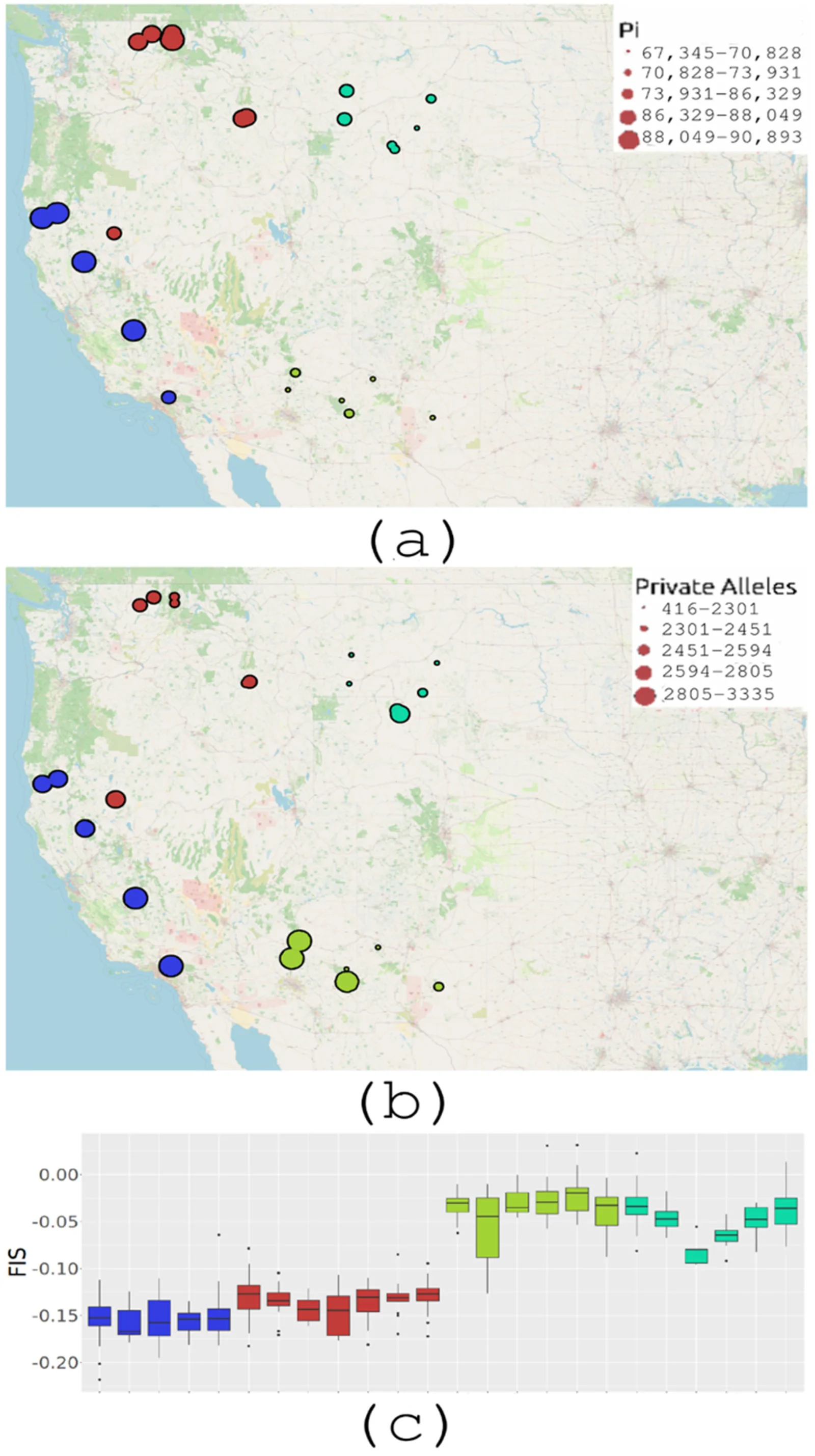
(**a**) Nucleotide diversity across seed sources in dataset two; legend shows how size of pixel relates to summed nucleotide diversity. (**b**) Private alleles across seed sources; legend shows how the size of pixel relates to the number of private alleles. (**c**) Inbreeding coefficient (Fis) across seed sources ordered West to East in dataset two. Colors are based on genetic clusters determined by the DAPC in Figure 1.

Population genomic metrics support broad scale patterns seen in population structure and suggest unique nucleotide diversity and private alleles occurring within genetic clusters (Figure 2). Nucleotide diversity and private alleles varied widely across sampled locations (Figure 2). Californian populations had consistently high nucleotide diversity and private alleles (Figure 2). Similarly, most populations in the northern coastal cluster areas had high nucleotide diversity and private alleles, with a few exceptions. Populations in the interior cluster and northern interior cluster showed the largest amount of within cluster variation in both nucleotide diversity and private alleles, with some populations in those clusters having some of the highest nucleotide diversity and private alleles and others the lowest magnitude in each metric. Both the California and northern coastal clusters show similar levels of inbreeding, with a large amount of excess heterozygosity (Figure 2). The interior and northern interior clusters also show generally similar levels of inbreeding but have less of an excess of heterozygosity compared with coastal clusters.

Geographic clustering patterns seen in pairwise Fst are further supported by significant isolation by distance (r = 0.6606423, permuted *p*-value = 1 × 10^−4^) (Supplementary Materials Figure S3). Environment also plays a role in genome differentiation, as seen by significant correlations found in the isolation by environment test (r = 0.234628, simulated *p*-value = 1 × 10^−4^) (Supplementary Materials Figure S3).

Two-dimensional site frequency spectra (2D SFS) support genome-wide differentiation seen in the DAPC and pairwise Fst, suggesting unique allele makeups across genetic clusters. More specifically, our results suggest that many high frequency alleles in interior genetic clusters occur at much lower frequency or are absent in coastal clusters and vice versa. This is seen via the many sites that occur at very different allele count classes between interior clusters and coastal clusters. Less pronounced differences are seen when the northern coastal cluster is compared with the California cluster, and when the interior cluster is compared to the northern interior cluster (Supplementary Materials Figure S4). These 2D SFS (Supplementary Materials Figure S4) still show differences in allele counts classes, supporting the pairwise Fst and DAPC clustering (Figure 1, Supplementary Materials Figure S2).

### 2.3. Demographic History

**Figure 3:**
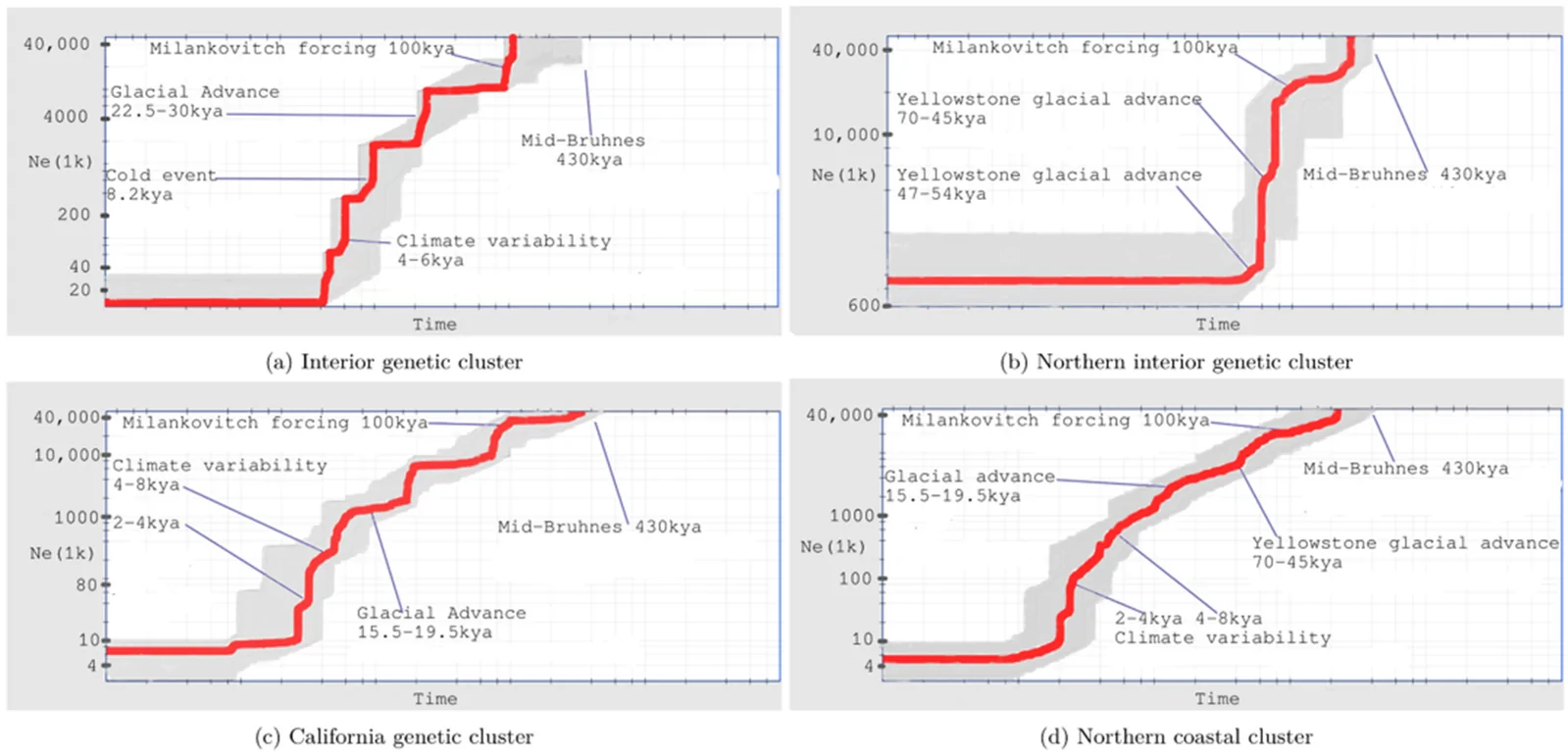
Modeled change in effective population size over time (log scale) from stairway plot for each genetic cluster found in dataset two. Red lines indicate mean estimates, while gray lines indicate 90,75,25,10 percent quantiles, respectively. Overlaid are Ne estimates following major bottleneck events. Full detailed plots of demographic history for each genetic cluster are found in Supplementary Materials Figures S5–S8. (**a**) Starting median estimates of Ne for the interior cluster is ca. 40,000,000 while current median Ne is ca. 20,000. (**b**) Starting median estimates of Ne for the northern interior cluster is 45,000,000 while current median Ne is ca. 900,000. (**c**) Starting median estimates of Ne for the California cluster is ca. 40,000,000 while current median Ne is ca. 8500. (**d**) Starting median estimates of Ne for the northern coastal cluster is ca. 40,000,000 while current median Ne is ca. 5500.

One-dimensional site frequency spectra (1D SFS) show an abundance of rare alleles within each genetic cluster. Stairway plot 2 suggests numerous bottlenecks for all four genetic clusters (Figure 3). Stairway plot 2 was able to estimate Ne for all clusters dating back to 400 to 500 kya. Starting Ne was similar for all clusters (slightly greater than 40,000,000), however final Ne estimates and the demographic histories varied for each cluster (Figure 3, Supporting Figures S5–S8). Even though the interior cluster is similar to the northern interior cluster (based on the DAPC, Fis, and pairwise Fst), the Interior genetic cluster shows a vastly different demographic history with multiple population bottleneck events occurring 4000 to 100,000 years ago (Figure 3, Supplementary Materials Figure S5). The northern interior genetic cluster shows the oldest population bottleneck with a long consistent decline in effective population size occurring between 50,000 and 300,000 years ago (Figure 3, Supplementary Materials Figure S6). Even though the northern coastal cluster and the California cluster are genetically distinct, they show similar demographic histories, with population bottleneck events occurring from 1000 to 100,000 years ago (Figure 3, Supplementary Materials Figure S7). The main difference between the demographic histories of the two coastal clusters is that the northern coastal cluster shows one long consistent decline in effective population size, whereas the California cluster shows multiple bottlenecks and plateaus over that time period (Figure 3, Supplementary Materials Figure S8).

### 2.4. Patterns of Selection

All three methods used to make inferences on selection showed similar results. General patterns for each metric were similar across genetic clusters, with the exception of Tajima’s D for the northern coastal cluster. ω = dN/dS and Ts/Tv were the same across genetic clusters, whereas Tajima’s D values varied across genetic clusters. A total of 9171 unique coding sequences contained polymorphisms; 73.94% had a dN/dS > 1, 18.65% had a dN/dS < 1, and 7.41% had a dN/dS equal to one. For Ts/Tv ratios, 97.54% of SNPs existed in windows greater than the equilibrium value.

The northern coastal cluster showed the highest number of SNPs that occur in windows with Tajima’s D within the simulated critical bounds and below the lower critical value, followed by a smaller number of SNPs that occurred in windows above the higher critical value (Table S3). The other three genetic clusters showed similar mixtures of SNPs occurring in windows above, within and below their respective critical values (Table S3).

### 2.5. Genomic Offset

**Figure 4:**
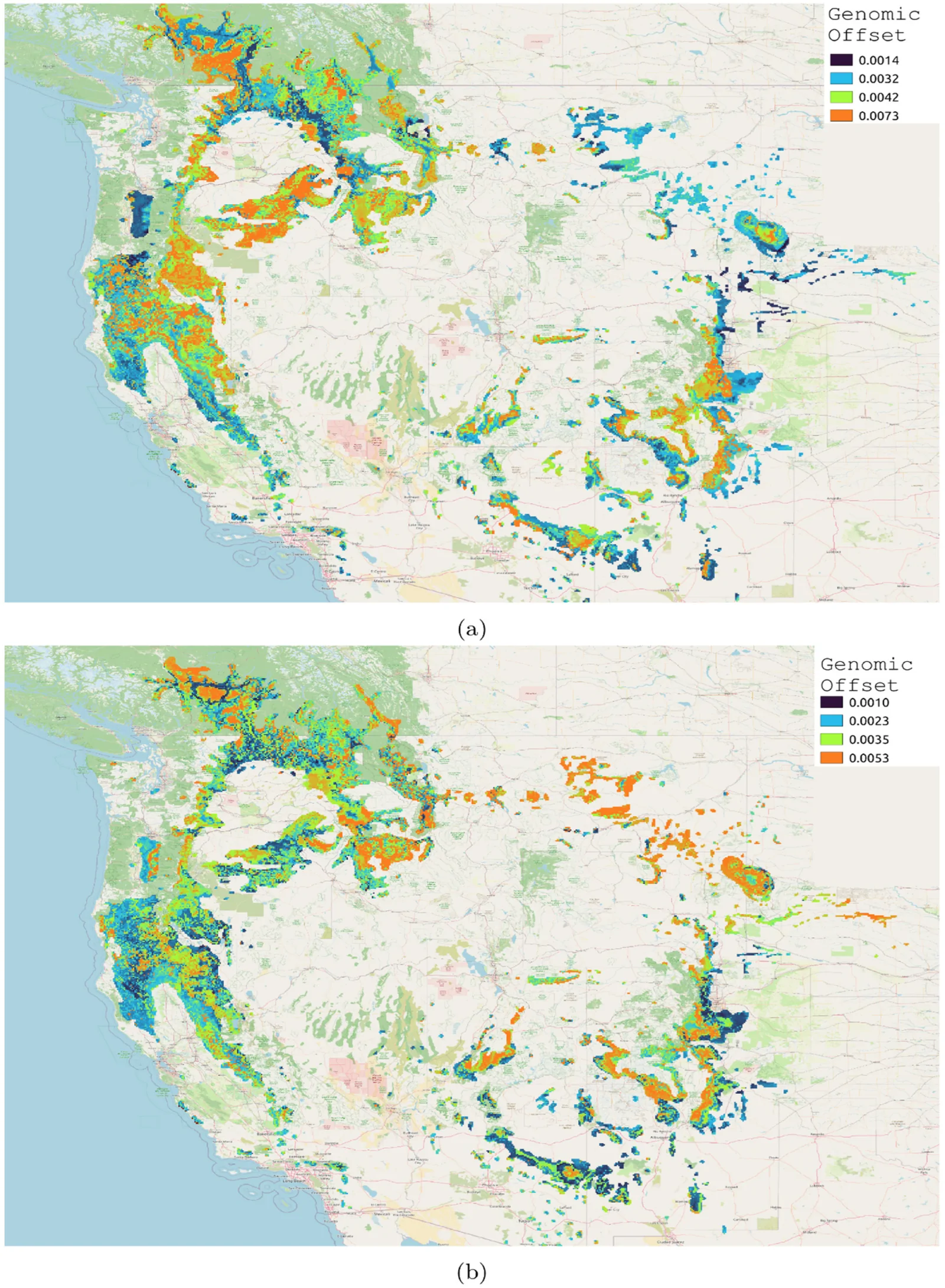
(**a**) Genomic offset predictions for 2041–2070 SSP 245 based on dataset one. (**b**) Genomic offset predictions for 2041–2070 SSP 245 based on dataset two. Darker orange pixels indicate higher genomic offset values, while cooler blue pixels indicate closer to zero genomic offset. Respective legends for specific genomic offset values and color relationships are shown in the top right of each figure.

Our genomic offset analysis identified many climate variables that are related to population structure (Supplementary Materials Figure S9, Tables S4 and S5) and showed that some areas are at high risk of maladaptation, whereas other areas are at very little risk. Current observed population structure was modeled using current climate data (Supplementary Materials Figure S10, Supplementary Materials Tables S4 and S5), suggesting the climate variables used in the analyses are important for population structure. Modeling the current observed population structure also provided a robust initial genotype by environment coordinate for calculating genomic offset, which helped support the robustness of the projections. Dataset one forecasts higher risk of maladaptation in portions of the four corner states, the northern Sierra Nevada, British Columbia, and eastern Oregon, based on the SSP 245 climate scenario in 2041–2010 (Figure 4a). In comparison, dataset two forecasts British Columbia, southern Idaho, portions of the four corner states, all of Montana and Wyoming and the Great Plains as areas with the highest maladaptation risk (Figure 4b). While there are similar broad patterns in areas of the range, the two datasets showed little congruence with a correlation value of −0.002 at the pixel level.

### 2.6. Genome by Environment Association (GEA)

Our analysis identified many candidate loci that are correlated to spring, summer and winter moisture. Genome–environment relationships were best shown by the sparse partial least squares regression (sPLS) compared to the redundancy analysis (RDA), suggesting stronger evidence of association of the loci identified using the sPLS. The candidate loci identified from the sPLS have biological functions related to water stress and developmental pathways, further supporting the role of these genes in environmental responses. For dataset one, 4711 SNPs were associated with summer precipitation, winter precipitation, and continentality (TD) and produced an adjusted R2 of 0.102. From those SNPs, 1426 were associated with summer precipitation, 1159 with winter precipitation and 2126 with TD. When using summer precipitation, winter precipitation and spring relative humidity, a similar adjusted R2 of 0.102 was observed and significant 3288 SNPs were identified. From those SNPs, 1249 were associated with summer precipitation, 922 with winter precipitation and 1117 with spring relative humidity.

For dataset two, 3147 SNPs were associated with summer precipitation, winter precipitation, and TD, produced an adjusted R2 of 0.08 and identified 9762 candidate loci. A total of 2787 SNPs were significantly associated with summer precipitation, 3347 were significantly correlated with winter precipitation and 3628 SNPs were correlated with yearly TD. When spring relative humidity, summer precipitation and winter precipitation were used in the RDA, an adjusted R2 of 0.09 was observed and 8854 candidate SNPs were identified. A total of 2153 SNPs were correlated with summer precipitation, 2835 SNPs were correlated with winter precipitation, and 3866 SNPs were correlated with spring relative humidity.

For dataset one, when all SNPs and all environmental variables were used the sparse partial least squares regression derived functions that explained an average of 15.32% of variance in the SNP matrix and an average of 29.5% of variation in the environmental data. The top one percent contributions of loadings for the original sPLS components consisted of 6672 SNPs and thus were chosen as candidate loci. Elevation and winter precipitation were the two variables in the 1% quantile of loadings for environmental data and thus chosen as the two variables with the most explanatory power for dataset one.

For dataset two, when the sPLS was run with all SNPs and all environmental variables, the sPLS functions explained 11.65% of variation in the SNP matrix and 19.17% of variation in the environmental data. 6246 SNPs in the second dataset were in the 1% quantile of loadings for this original sPLS function and thus were chosen as candidate loci. Spring relative humidity and summer precipitation were the two environmental variables in the 1% quantile for the environment data and therefore chosen as the two most explanatory environmental variables for dataset two. Due to the higher variance explained by the sPLS for both datasets, the candidate loci identified from sPLS for both datasets were used for further analysis via functional annotation.

**Figure 5:**
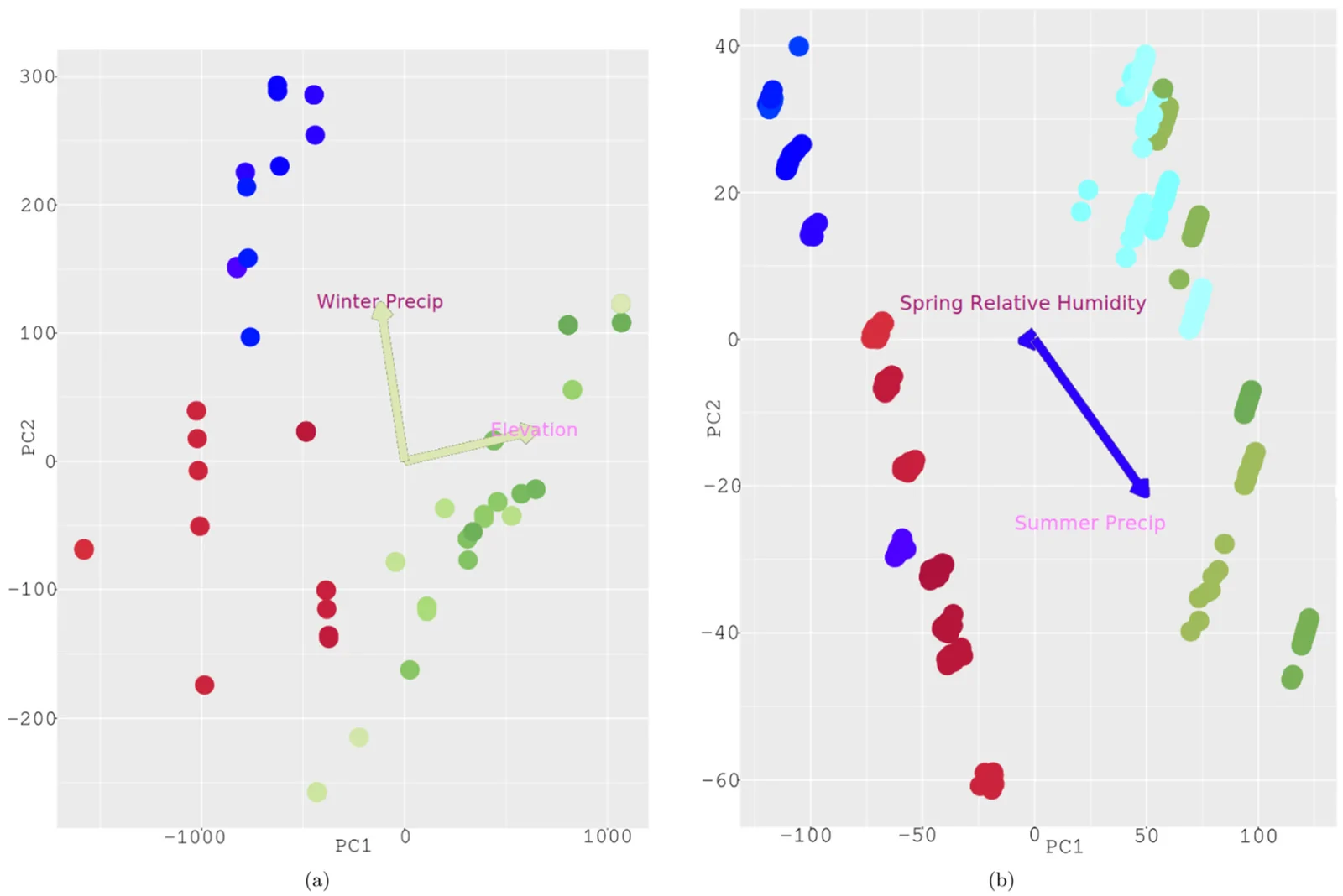
Biplot of candidate SNPs and important environmental variables denoted from the sPLS. Individuals are shown colored by the genetic cluster from Figure 1 and Figure 2. Greens represent the interior cluster, cyans represent the northern interior cluster, reds represent the northern coastal cluster and dark blues represent the California cluster. In dataset one, PC 1 accounts for 96.19 percent of variation and PC 2 accounts for 3.57 percent of cumulative variation. In dataset two, PC1 accounts for 73.94 percent of variation, and PC2 accounts for 11.25 percent of cumulative variation. Vectors represent where specific variables increase, that is, following the vector from 0 outward each variable goes up while on the other side of the line the variable goes down. (**a**) Allele changes in response to increasing elevation are seen left to right, while allele frequency changes in response to increasing winter precipitation are seen top to bottom; (**b**) shows how allele frequency changes in response to increasing spring relative humidity are seen right to left in the plot. Allele changes in response to increasing summer precipitation are seen along a diagonal top left to bottom right in the figure.

A PCA of candidate loci for each dataset along with their respective climate variables is shown in Figure 5. Each PCA shows stark differentiation among genetic clusters and some within genetic cluster variation. These show that the alleles that occur in the California cluster are generally associated with higher winter precipitation and spring relative humidity, but lower summer precipitation (Figure 5). The northern coastal cluster has alleles associated with increasing winter precipitation, summer precipitation and elevation but relatively similar spring relative humidity (Figure 5). The southwest and Great Plains cluster also has alleles that are associated with a gradient of winter precipitation, summer precipitation and elevation and an overall lower spring relative humidity compared to coastal clusters (Figure 5). Interestingly, CA 993 clusters closer to the northern coastal cluster sources in these candidate loci, but when all SNPs are considered the seed source clusters with the California coastal cluster. This is a high-elevation seed source and may have a climate more similar to the northern coastal cluster rather than coastal California.

### 2.7. Functional Annotations

For dataset one, SnPeff identified that 93.217% of candidate loci had a modifier effect, along with another 4.361% having a moderate effect. 65.622% of SNPs were missense variants with another 31.734% being silent variants. 84.58% of candidate loci were in introns with 15.42% being in exons. There was an overall Ts/Tv ratio of 2.4307. Eggnog identified 1282 unique genes from gene prediction that were involved in 277 unique KO terms and 3124 unique GO terms. Twelve of those KO terms, and 157 GO terms were in the 95th percentile of occurrences or above (Table 1). Metabolic and environmental adaptation pathways were the common pathways of the most common KO terms (Table 1). The most common GO terms included response to abiotic stress, defense pathways, signal transduction, transcript regulation, metabolism and cell/organelle development. For dataset two, SnPeff identified 93.437% of candidate loci as having a modifier effect, along with another 3.934% having a moderate effect. Missense variants accounted for 61.011% of variants and 36.18% were silent variants; 83.41% of variants were in introns and 16.59% were in exons. The overall Ts/Tv ratio was 2.4252. Eggnog identified 948 genes from gene prediction that were involved in 212 unique KO terms and 2715 unique GO terms. Eleven KO terms and 135 GO terms were in the 95th percentile of occurrences or above (Table 1). Similar pathways and GO term functions to those observed in the first dataset were found among these most common terms for dataset two. Queries for all candidate loci, ortholog metrics, KO and GO terms are shown in Supplementary Materials, Table S6.

The most abundant *A. thaliana* orthologs for both datasets are shown in Table 2. Similar protein occurrences are seen across both datasets, showing consistency of sPLS across datasets. Genes producing non-specific serine/threonine protein kinase (EC 2.7.11.1), protein kinase domain-containing protein and RING-type E3 ubiquitin transferase (EC 2.3.2.27) were some of the most abundant orthologs identified. ABC transporter genes were also abundant. Other important orthologs included transcription factors such as Cytochrome P450, Heat Shock proteins, growth regulation factors, elongation factors, Myb DNA-binding, WRKY protein family, and regulation of telomere elongation factor helicase 1 (Supplementary Materials Table S6). The explained variance across SNP and environmental datasets from the sPLS along with the level of effect of the variants and the biological relevance of the most common KO terms, GO terms and *A.thaliana* orthologs, all support a strong association of the loci with the environmental variables identified.

## 3. Discussion

### 3.1. Genome-Wide Analysis Supports the Presence of Four Genetic Clusters Currently in the Species

This study suggests there is a need to update current variety distinctions in ponderosa pine. Our results support four distinct clusters within ponderosa pine: a California cluster, a northern coastal cluster, an interior cluster and a northern interior cluster. Our study shows the clearest differentiation between Montana and Wyoming and the rest of the intermountain west relative to previous reports [16,17,18,19,20,29]. The 2D SFS showed differentiation in composition of alleles between the interior and northern interior clusters, further supporting that they are indeed unique genetic clusters. Interestingly, our results somewhat mirror differentiation in the coastal portion of the ponderosa pine seen in previous studies [16,17] with the main difference in our results showing that California is differentiated from Washington, Oregon and Idaho. Genetic cluster designation is strongly supported by the model fit for the AMOVA, based on MSD terms for population and error terms. Our results provide the most comprehensive genome-wide study of population structure in ponderosa pine to date; however, phylogenetic analysis including an outlier species is needed to provide further support for the number of varieties in the species.

### 3.2. Bottleneck Events and Their Effect on Effective Population Sizes

The results of Stairway plot 2 suggest that broad and fine scale bottlenecks from the mid-Pleistocene to the Holocene played a major role in the strongly structured genetic clusters we see today. The extremely high effective population size that each genetic cluster had (40–50 million individuals) ca. 400–500 kya, would suggest much higher continuity in populations of ponderosa pine in the mid-Pleistocene. This continuity in ponderosa pine populations most likely lessened with each bottleneck, furthering differentiation across genetic clusters with each subsequent bottleneck due to random losses of alleles and reduced corridors for gene flow [2,30,31].

The specific palaeoecological events that are consistent with estimated population bottlenecks are as follows. The Mid-Bruhnes event occurred ca. 430 thousand years ago (kya) [32,33], our demographic modeling shows strong declines in the lower confidence bounds in all clusters around that time. A Milankovitch forcing event, characterized by a sudden cooling and warming based on the gain and loss of ice, is believed to have occurred around 100 kya [34]. Around this time, our demographic models show a strong decline in Ne followed by subsequent stability in the interior, California, and northern coastal clusters and a smaller decrease in effective population size in the northern interior cluster. A major Yellowstone glacial advance is estimated to have occurred between 70 and 45 kya [35]. In that time period, we see a sharp decline in effective population size of the northern interior cluster (the closest cluster to that glacier), a small decline in the California and northern coastal clusters but stability in the interior cluster. Another Yellowstone glacial advance was believed to occur between 47 and 54 kya [35], which coincides with another small decline in Ne in the northern interior cluster and a steady decline in the northern coastal cluster, but stability in the interior and California clusters, which are much further away from that glacial advance.

Glacial advances across the northern Rocky Mountains were also believed to occur between 34 and47 kya [35], which coincides with a continuing decline in the northern coastal cluster but stability in all other clusters. The stability of the northern interior cluster in this time, and from there on, may indicate that populations were existing in refugia with high and consistent levels of protection from other glacial advances and climatic events due to the strong die-off seen in previous events. Between 22.5 and 30 kya, a glacial advance occurred in the Rocky Mountains [35]. During that time, we see a sharp decline in Ne in the interior cluster, a consistent continuing decline in the northern coastal cluster, the beginnings of a decline in the California cluster and stability in the northern interior cluster. A glacial advance then occurred between 15.5 and 19.5 kya [35], during which we see the end of a sharp decline in the interior cluster, a sharp decline in the California cluster, and the continued consistent decline in the northern coastal cluster. A multi-millennia long drought is believed to have occurred from 14.5 kya to 12.9 kya [36]. During this time, we see a drop in Ne in the lower confidence intervals of the interior cluster, along with a strong drop in Ne that occurs after this time period in the mean projection. The northern coastal cluster also shows the continuing consistent decline during that time period. Interestingly, we see relative stability in the California and northern interior clusters across all other clusters during this time, which potentially suggests that not all portions of North America experienced that drought or that these regions were in a local refugia during this event. A cold event is thought to have occurred around 8.2 kya [37]. During that time, we see sharp die-offs in all clusters besides the northern interior cluster. From 4 to 8 kya, a general decline in temperature was combined with general increases in moisture [38]. During this time period, high variability in both temperature and moisture is still believed to have occurred [38]. Decreases in Ne in the 4–8 kya time period are observed in all clusters besides the northern interior cluster. During this cooler and wetter period, ponderosa pine might have been outcompeted by other faster-growing high-elevation conifers, causing this bottleneck event. Bottlenecks due to competition may be seen again between 2 and 4 kya, as there was another large increase in moisture and cooler temperatures [38]; we see relative stability in the interior and northern interior clusters but declines in both the California and northern coastal clusters. The stability seen in the interior cluster during this time period suggests that around 4 kya, the cluster reached its current occurrence, whereas the current occurrence for the California and northern coastal clusters may have not occurred until after this event ended around 2 kya.

### 3.3. Factors Shaping Current Population Structure

Our GEA analyses suggest that winter, spring and summer moisture availability act as the most important selection pressures that currently affect ponderosa pine populations. Moisture availability varies greatly among and within genetic clusters (Supplementary Materials Tables S4 and S5) [11]. Pairwise Fst (Supplementary Materials Figure S2) and DAPC (Figure 1) both suggest at least moderate levels of gene flow occurring within all genetic clusters, despite the stark environmental gradients. This gene flow across heterogeneous environments within clusters may have created an abundance of heterozygotes even with numerous bottleneck events [7,8,9]. We saw one high-elevation southern California seed source (CA 993) showing similar alleles across candidate loci as the northern coastal cluster (Figure 5). This could be due to similar climates independently favoring specific alleles [3,4,5,6], creating similar populations despite minimal chances of gene flow, or it could be due to a remnant of when California and northern coastal states had higher continuity before the numerous bottlenecks occurred. Future studies should examine the occurrence of selection over time using simulation methods.

While it is difficult to determine whether demographic events or selection alone created differentiation in specific loci, our results show the importance of the interplay between both demographic history and selection in shaping what we see today out on the landscape [1,2]. Furthermore, our results suggest that diversifying and balancing selection may maintain genetic variation across the species’ range, based on dN/dS, a high Ts:Tv bias and Tajima’s D. The different critical values seen in the northern coastal cluster in the Tajima’s D analysis highlight the sensitivity of that metric to demographic history, as similar start and end Ne’s are seen in that cluster, but the magnitude of bottlenecks is much smaller, creating a wider distribution of possible neutral values of Tajima’s D. Furthermore, our results confirm the importance of demographic history in making inferences about selection, as our Tajima’s D results would be very different if we did not create unique neutral Tajima’s D distributions for each genetic cluster and relied on the unit normal distribution, N(0,1), that Tajima’s D theoretically approximates [39].

### 3.4. Ecological Implications of GEA Candidate Loci

The importance of moisture availability in tree health is well established, and our results suggest that moisture variables would be an important factor to keep in mind for assisted gene flow or assisted migration [40]. More specifically, winter and summer precipitation and spring relative humidity, along with continentality, were important variables in the GEA and gradient forest analyses. California and the northern coastal individuals are associated with higher spring relative humidity but lower summer precipitation. The northern coastal cluster are associated with higher winter precipitation. The interior and northern interior clusters are associated with a range of summer precipitation values but lower spring relative humidity. These patterns of association are seen in the changes in allele frequency in response to changing climate variables (Figure 5), which supports the idea that certain alleles are successful under specific moisture conditions [3,4,5,6]. Many of the functions identified in our GEA orthologs were also identified as important genes in drought and water use efficiency traits of ponderosa pine in previous studies [41], supporting the biological relevance of our GEA loci. Our study provides a foundation for future studies to explore if these allele and climate associations offer robust indicators of seed source transfer performance in restoration settings.

### 3.5. Increased Risk of Maladaptation Evidenced by Genomic Offset

Our genomic offset revealed that all genetic clusters have areas at risk of maladaptation, most likely due to drastic shifts in future climate. Both models predict high risk of maladaptation in the northern coastal cluster, especially central Idaho and British Columbia; the inter-basin in the four corners states, especially in Colorado and northern New Mexico; and northeastern California. The Great Plains show minimal agreement between the two models, and as such, the risk of maladaptation is more uncertain but adverse effects should be monitored. Many areas with high maladaptation risk coincide with recent studies showing altered survival, growth and phenology in response to changing climate [42], increased mortality events [27,43], and reductions in growth, regeneration, and seedling survival, as a consequence of climate change and other compounding factors such as bark-beetle outbreaks and higher intensity fires [26,27,28]. The predicted species-wide risk of maladaptation is greatly exacerbated by the rapid and drastic changes in winter and summer moisture and spring relative humidity [44]. As the climate continues to change, seed sources can be picked to match newer climate regimes based on our GEA results. The results of our genomic offset analyses located the most at-risk ponderosa populations across the species’ distribution, providing key information for conservation and management practices.

The disparity in the two models at the pixel and landscape levels shows the importance of training data in making model projections, as dataset one did not have any samples from Montana and Wyoming, which is a potential reason why the projections are different. Other reasons could be due to the study design, as dataset one came from mature trees in natural populations and dataset two came from seedlings in a greenhouse. Many seedlings grown in controlled conditions may not survive to adulthood, whereas mature trees have gone through strong selection filters and survived climatic changes in their lifetimes, providing a more accurate signal of climate adaptation. Further studies should include field validation of forecasted areas with high maladaptation risk from both datasets.

Genomic offset is a relatively new method and a rapidly developing field of study that still needs field-based tests to confirm model projections, even with all due diligence conducted by investigators. The overlap in predicted maladaptation and negative effects seen in field surveys support our projections [26,27,28,42,43]. Higher genomic offsets have also been correlated with negative effects in common gardens in other systems [45], suggesting that this method provides robust inferences that might require field validation.

## 4. Materials and Methods

We used two independent genomic datasets, a pooled sampled dataset and an individual level dataset. For population structure, population genomic metrics, demographic history and patterns of selection, we used only the individual level dataset. This was done out of an abundance of caution, as the interpretation of many of these metrics is rooted in samples being individually sequenced. We used both datasets to assess genome by environment relationships and genomic offset.

### 4.1. Dataset One (Pooled Samples) Sample Collection

Needle tissue was collected from 12 to 20 mature individuals in 26 unique locations across the species’ natural range (Supplementary Materials Table S4). Sampled individuals were at least 30 m from each other, to reduce the probability of sampling close relatives. The terminal end of a branch was removed, and 5 to 10 fascicles of needles were collected from each individual tree. Latitude, longitude, and elevation were recorded for all individuals. Following collection, all needles were stored in a −20 °C freezer. All 26 locations were on public lands managed by the United States Forest Service (USFS) or the United States Bureau of Land Management (BLM). Most collections were conducted by respective USFS, BLM or Cal Fire personnel; any collections not conducted by USFS or BLM members were conducted with the approval of each respective organization. All 26 locations represented novel ponderosa pine sampling locations with no known official seed source name and, as such, short-hand names were arbitrary.

### 4.2. Dataset Two (Common Garden) Sample Collection

Seeds were collected from 24 seed sources across the geographic range of ponderosa pine and divided into four regions, with six seed sources coming from each region: (1) eastern Washington State and Idaho; (2) Arizona, Southwestern Utah and New Mexico; (3) Montana and Wyoming; (4) California (Figure 1, Supplementary Materials Table S5). Seed sources were selected based on availability from previous collections maintained by Northern Arizona University (NAU), The Forest Service’s Experimental Forest at Priest River, Idaho and the Forest Service’s National Seed Laboratory. Seed-source names followed the names assigned by the original collectors and were retained for consistency. A total of 50 to 60 seeds per seed source were sown in November 2022 in the Northern Arizona University Greenhouse. Seed was removed from the freezer, sown and watered in on the same day. Germinated seedlings were watered to saturation three times a week and housed in 20 cm diameter pots for the duration of this experiment. Seedlings were also fertilized to maintain health and growth throughout their first year. Tissue for DNA extraction was collected from 404 seedlings (101 individuals per region, 5–32 individuals per seed source).

### 4.3. DNA Extraction and Sequencing

For both datasets, DNA was extracted using 60 to 100 mg of needle tissue homogenized using a SPEX Tissue MiniG 1600 Prep Machine^®^. DNA was extracted using either a MP Bio EZ Plant DNA extraction kit (MP Biomedicals, LLC, Irvine, CA, USA), Nucleospin Plant DNA extraction kit (MACHEREY-NAGEL, Dueren, Germany) or a Cetyltrimethylammonium bromide (CTAB) DNA extraction protocol. Following extraction, each extract was quantified using a Qubit 4.0 Fluorometer (Thermo Fisher, Walthman, MA, USA) and stored in a −20 °C freezer. For dataset one, DNA samples were pooled in sets of 4 with respect to their sampling location, creating 96 composite samples. Equal amounts of input DNA from each individual were combined so that each sample was equally represented. Two samples failed to produce viable libraries, leaving 94 composite samples to sequence. No pooling was conducted for dataset two; all 404 individuals were sequenced individually. All samples across the two datasets were then sequenced using the same 55,000 capture-seq probes (40,000 probes for coding regions, 15,000 for intergenic/non-coding regions). Probes were designed using a draft ponderosa pine transcriptome (Gilewski and Mattsson, unpublished data) and the loblolly pine (*Pinus taeda*) reference genome v 2.0 (https://treegenesdb.org/FTP/Genomes/Pita/v2.0/genome/, accessed on 23 June 2023). Libraries were sequenced using 150 × 150 bp on an Illumina platform conducted at Rapid Genomics (LGC Biosearch Technologies, Gainesville, FL, USA). Probes generated 300–400 Mbp of data per sample.

### 4.4. Sequence Analysis

The same software for sequencing analysis was used for both datasets; however, each dataset was analyzed independently. Raw reads were quality controlled and trimmed of adapters using fastp [46] (v0.23.2), FASTQC (v0.12.1) [47] and MultiQC (v1.14) [48]. Initial filtering criterion for raw reads was a Phred score higher than 30. Following initial QC, reads were mapped to the loblolly pine reference genome (version 2.0) using bwa-mem [49] (v0.7.10) using default settings. Mapped reads were then sorted by coordinates and any duplicates were removed using samtools [50] (v1.5). Following mapping, single nucleotide polymorphisms (SNPs) were called using bcftools [51]. For dataset one, 111,576,152 SNPs were initially called. For dataset two, 284,913,192 SNPs were initially called. Initial filtering criteria for SNPs were quality score greater than 20, call rate greater than 0.75, and minor allele frequency (MAF) greater than 0.01. SNP filtering was conducted using vcftools [52] and bcftools [51]. After initial filtering, 1,282,955 SNPs and 1,501,748 SNPs (datasets one and two, respectively) were retained for further analyses.

### 4.5. Current Population Structure

Population structure was assessed using a principal component analysis (PCA) and a discriminant analysis of principal components (DAPC), with functions within plink [53] and adegenet R package (v2.1.7) [54]. The optimum number of PCs included in the DAPC was selected based on the optim.a.score function within adegenet. Twenty discriminant axes were included in dataset two; the optimum number of PCs for dataset two in the DAPC was 12 with a classification accuracy of 76%. The effect of markers potentially under selection on population structure was assessed by re-running all analyses using putative-neutral markers, as determined by Hardy–Weinberg equilibrium (HWE) using vcftools (v0.1.16) [52]. The markers potentially under selection did not have any effect on population structure and so the results of the full datasets are presented in the main text (Supplementary Materials Figure S1). K means clustering, which used eigenvectors from the PCA and BIC as the evaluation metric, was conducted using adegenet [54]. The max number of K’s possible was limited to the number of collection locations, i.e., 24 for dataset two. Wright’s pairwise fixation index (Fst) [55] was calculated as an additional test of differentiation. Pairwise Fst was calculated using the R package StAMPP (v 1.6.3) [56] and visualized using a neighbor joining tree from ape (v 5.8.1) [57]. An analysis of molecular variance (AMOVA) was conducted using StAMPP [56]. Grouping factors for the AMOVA were based on the clustering seen in all previous analyses and were set to four for dataset two. Each AMOVA was conducted with 1000 permutations.

### 4.6. Current Population Genetic Metrics

Nucleotide diversity *ϴπ* [58], inbreeding coefficient (Fis) and private alleles were calculated on putative-neutral markers. To identify putatively neutral markers, a HWE alpha value of 0.05 was used, leaving 1,018,650 SNPs. *ϴπ* was calculated using 10 kb windows of each scaffold using vcftools [52]. *ϴπ* and private alleles for each collection location were calculated using functions found within snpR (v 1.2.14) [59]. Fis was calculated using vcftools [52,60].

Isolation by distance (IBD), isolation by environment (IBE) and site frequency spectra (SFS) were estimated using neutral and non-neutral markers. IBD and IBE were conducted using Mantel tests with 9999 permutations with functions within the ade4 R package (v 1.7.24) [61]. Latitude and longitude were used for IBD analysis, and 33 climate variables were used for IBE (Table 3, Supplementary Materials Tables S4 and S5). SFS was calculated using R scripts from [62]. 1D SFS were generated for each genetic cluster. 2D SFS were then generated for pairwise combinations of genetic clusters within each dataset.

### 4.7. Future Maladaptation Risk Using Genomic Offset

Both full SNP datasets (neutral and non-neutral markers) were filtered using a higher MAF filter (0.05) for genomic offset. This higher MAF left 677,119 SNPs for dataset one and 649,507 SNPs for dataset two. Missing SNPs were imputed by filling in missing values for each site with the most common value for each site. Climate data was downloaded from ClimateNA (v7.42) [63,64]. Genomic offset was estimated using gradient forest (v 0.1-37) [65]. Genomic offset is a metric used to assess risk of maladaptation in the future based on how much the genomic composition would have to change in order to have the same genome by environment relationships seen today. That change is measured based on the distance between the current and future genome by environmental coordinate spaces that gradient forest creates. Each dataset was tested independently using the same approach and the final inferences from each model’s projections were compared. Training consisted of fitting the gradient forest model to observed SNPs and climate data. Model quality was assessed by seeing if the model could accurately project range-wide population structure after training. This was done by conducting principal component analysis on the current genome by environment coordinate space and converting the top 3 PCs to RGB values. This is one way to make sure the current reference point of the current genome by environmental space mirrors patterns seen on the landscape. The final model for both datasets consisted of 33 climate variables, 1000 trees and 101 bins. After training on each dataset and calculating the current genome by environmental space, the future genome by environmental space was predicted for the shared socioeconomic pathway (SSP) 245 climate scenario for 2041–2070 using the 8 global circulation model ensemble model projections from ClimateNA [63,64]. Genomic offset was then calculated by taking the Euclidean distance between the current genome by environment space and the future genome by environment. To examine relationships between the models, correlations between dataset one and dataset two genomic offset values were calculated using base functions in R (v4.4.2) [66]. Genomic offset predictions were visualized using qGIS (v3.22) [67].

### 4.8. Genome by Environment Relationships Using Genome by Environment Association (GEA)

The same imputed SNP datasets from the genomic offset analysis were also used for GEA analysis. Selection of environmental variables included in the GEA was based on gradient forest importance measure. Because redundancy analysis (RDA) is sensitive to collinearity among variables, a sub-selection of variables from the genomic offset were used with the RDA, based on variable importance metrics found within gradient forest. Gradient forest identified summer precipitation, winter precipitation and yearly continentality as the three climate variables with the highest importance in dataset one, and summer precipitation, winter precipitation and spring relative humidity as the three variables with the highest importance in dataset two. To account for all potential combinations of variables, datasets one and two were run using both sets of climate variables in separate tests. The RDAs were conducted, and overall model fit was assessed using functions within the vegan (v2.7.5) R package [68].

Sparse partial least squares regression (sPLS) was also used for GEA, using functions within the RGCCA R package (v3.0.3) [69]. sPLS was run with default parameters. For both datasets, initially all SNPs and all environmental variables from the genomic offset analyses were tested. All environmental variables were used for the sPLS, because sPLS relies on an eigen decomposition of matrices to then create functions that maximize variance among matrices; it is therefore less sensitive to unstable parameter estimation among variables due to the effect of collinearity relative to the multiple linear regression used in the RDA. Following these full model tests, the loadings for the respective sPLS functions for the SNP matrix and the environmental matrix were assessed. The variables that were in the 1% quantile of loadings in both the SNP dataset and climate variable dataset respectively were chosen as candidate loci and important environmental variables.

Following the identification of candidate loci, both RDA and sPLS were then compared using variance explained (R2), to see which model best explained the SNP data and thus gave the most robust representation of environmental candidate loci. A PCA was then re-run, using FactoMineR (v2.17) [70], on candidate loci and the most important environmental variables to illustrate how alleles changed with respect to changing climate.

### 4.9. Functional Annotation of Candidate Loci and Enrichment Analysis

A custom database for the loblolly pine (*Pinus taeda*) reference genome was created with SnpEff (v5.2) [71]. The genes for SNPs identified from the GEA were identified using the Pita 2.01 annotation. Sequences for all candidate loci were then extracted from the reference genome using samtools [49] (v1.5). Functional annotation was first conducted on candidate loci using eggnog (v2.1.12) [72]. To gain more information on functions of candidate loci, *A. thaliana* orthologs were identified using the blastx function on NCBI with search restricted to *A. thaliana*. Unique GO and KO terms were identified for candidate loci for both datasets. The counts of each GO and KO term were used to create a distribution of observed occurrences to identify the most common genes. Any term that was in the 95th percentile or greater was retained for further analyses.

### 4.10. Demographic Modeling and Patterns of Selection

Demographic history was inferred using changes in effective population size (Ne) using Stairway plot 2 (v2.1.3) [73]. One-dimensional SFS with singletons excluded for each unique genetic cluster in dataset two were used. Stairway plot 2 is very sensitive to singletons. Only dataset two was used in this analysis as there was a concern that using pool-seq data may provide false inferences due to the very different distributions of 2D SFS in dataset one compared to dataset two. Stairway plot 2 has only been used for diploid samples in previous studies. Twenty years was used as the estimated generation time and 1.0 × 10^−9^ was used as the estimated mutation rate per generation based on a midpoint value within the range of nuclear mutation rates of pines 0.70–1.31 × 10^−9^, found in [74], which also falls within the evolutionary rate estimated for gymnosperms in [75].

Signatures of selection were inferred using multiple methods; (1) ω = dN/dS, (2) transition-to-transversion ratios (Ts/Tv), and (3) Tajima’s D that was corrected for demographic history using simulations. These analyses were limited to only dataset two as all metrics were originally designed for individually sequenced samples. Because identifying selection is so nuanced, patterns from all three methods were used to make final inferences on signatures of selection. ω = dN/dS, was estimated by dividing non-synonymous mutations by synonymous mutations for each coding sequence identified using SnPeff [71,76,77]. Our reference genome is from a different species, and thus we are comparing interspecific sequences to calculate dN/dS, following suggestions seen in the previous literature [78,79]. Ts/Tv was calculated on 10 kb windows using vcftools [52]. High Ts/Tv bias has been related to candidate genes in many species [80,81,82] but is by no means a perfect signature of selection. Tajima’s D is a classic test of neutrality that is extremely sensitive to demographic history [39]. To correct Tajima’s D for demographic history, first median estimates of Ne across generations for each genetic cluster were used in SLiM 3 (v5.2) [83] to create neutral simulations that corrected for each genetic cluster’s specific demographic history. Simulations were conducted on 100 MB fragments using only fixed effect neutral mutations (selection coefficient = 0.0) and the same mutation rate (0.70–1.31 × 10^−9^) used in stairway plot 2 models. Recombination rate of 10^−4^ was used for all simulations, which is based on a rounded lower bound of conifers’ genome-wide recombination rates from [84]. Twenty independent simulations were run for each genetic cluster. Tajima’s D was then calculated on simulated VCFs in 10 kb windows using vcftools [52] creating 100 Tajima’s D windows that represented possible Tajima D values for neutral mutation; the minimum and maximum simulated values were used as upper and lower critical values for rejecting the null hypothesis of Tajima’s D. The critical Tajima’s D values for the interior cluster were −2.1451 < neutral < −0.951064. The critical Tajima’s D values for the northern interior cluster were −2.27033 < neutral < −0.892826. The critical Tajima’s D values for the northern coastal cluster were −1.52952 < neutral < 0.813934. The critical Tajima’s D values for the California cluster were −2.29491 < neutral < −0.951064. Following the simulations, observed Tajima’s D values were then calculated in 10 kb windows for each individual genetic cluster using vcftools [52]. Any observed Tajima’s D value below the lower critical value obtained from simulations was considered to be under purifying selection; similarly, any observed Tajima’s D value above the higher critical value for each genetic cluster was considered to be under balancing selection.

## 5. Conclusions

In summary, genome-wide data suggests the need to revise the current number of varieties in ponderosa pine. We found strong evidence of demographic events and selection shaping population structure. Moisture-related variables were the most important selection pressures causing differentiation.

## Figures and Tables

**Table 1:** The most common KO terms for dataset one and dataset two. For terms that occurred in both datasets, the number of occurrences follows dataset order, that is the first number is the occurrence in the first dataset, and the second number is the occurrences in the second dataset. KO pathway indicates what KEGG orthology pathway the term fits into. Description represents biological functions of each KEGG term.

KEGG KO Identifier	Dataset One or Dataset Two	Number of Occurrences	KO Pathway	Description
K01179	Dataset one and 2	11, 7	Metabolic pathways	Sugar Starch Metabolism, Signal transduction, hydrolases
K00128	Dataset two	7	Metabolic Pathways	Metabolism of lipids, sugars proteins. Oxoreductases, aldehydrogenases
K10529	Dataset two	7	Metabolic pathways	Metabolism of lipids and secondary metabolites
K15639	Dataset two	10	Metabolic pathways	Metabolism of terpenoids and polyketides
K21374	Dataset two	8	Metabolic pathways	Metabolism of terpenoids and polyketides
K00430	Dataset two	17	Metabolic pathways	peroxidases, Phenylpropanoid biosynthesis, Biosynthesis of secondary metabolites, oxidoreductase
K04733	Dataset one and two	19, 13	MAPK signaling pathway	Environmental Information Processing; Signal transduction
K05658	Dataset one and two	14,14	ABC transporters	ATP hydrolysis, transport of a wide variety of substrates such as ions, sugars, lipids, sterols, peptides, proteins
K05666	Dataset one and two	18,14	ABC transporters	ATP hydrolysis, transport of a wide variety of substrates such as ions, sugars, lipids, sterols, peptides, proteins
K05670	Dataset one	9	ABC transporters	ATP hydrolysis, transport of a wide variety of substrates such as ions, sugars, lipids, sterols, peptides, proteins
K05909	Dataset one	14	Unclassified: metabolism	Oxidoreductases
K08235	Dataset one	10	Unclassified: metabolism	Transferases
K09422	Dataset one and two	32,13	Protein families: genetic information processing	Transcription factors
K14638	Dataset one and two	10,8	Protein families: signaling and cellular processes	Solute and peptide carrier family
K16297	Dataset one	12	Metabolic pathways	Peptidases and inhibitors
K20279	Dataset one	9	Metabolic pathways, Environmental Information Processing, Protein families: genetic information processing	Carbohydrate metabolism, signal transduction, membrane transport, hydrolases
K10398	Dataset two	8	Cellular Processes	Centrosome formation proteins, Spindle formation proteins
K21867	Dataset one	10	Protein families: signaling and cellular processes	Ion channel Transporters

**Table 2:** Orthologs of candidate loci identified using the sPLS in the GEA not listing hypothetical proteins, which occurred 493 times and uncharacterized proteins which occurred 204 times. Gene Names shows the 4 genes that occurred the most which produce each respective protein. Number of loci columns indicates the number of candidate loci that can produce each specific protein. Biological process column shows the most common biological functions for each protein.

Protein	Gene Names	Number of Loci in Dataset One	Number of Loci in Dataset Two	Biological Process
non-specific serine/threonine protein kinase (EC 2.7.11.1)	At3g61160, At4g03230, At1g29730, At1g07650	268	247	Regulators of GSK3s; Development and drought signaling
Protein kinase domain-containing protein	At1g17230, At4g32000, AXX17_At2g41210, AN1_LOCUS10399	169	155	Plant growth and stress response trade-offs, Environmental stress response
RING-type E3 ubiquitin transferase (EC 2.3.2.27)	At1g68940, At2g19410, At3g49060, At5g61560	212	127	Adaptation to soil conditions, Salt and drought stress response
Protein kinase superfamily protein	At2g25220, At4g13020, At1g24030, At1g71530	120	123	Environmental stress response
Glycosyltransferase (EC 2.4.1.-)	AN1_LOCUS10132, AN1_LOCUS10133, AN1_LOCUS12454, AN1_LOCUS13037	110	102	Heat, drought and salt stress responses
glucan endo-1,3-beta-D-glucosidase (EC 3.2.1.39)	At5g24318, At1g11820, At1g30080, At2g01630	78	78	Stomatal opening, Drought response
Peroxidase (EC 1.11.1.7)	AN1_LOCUS13615, AN1_LOCUS18912, AN1_LOCUS19344, AN1_LOCUS19347	72	72	Temperature and water stress response
ADP-ribosyl cyclase/cyclic ADP-ribose hydrolase (EC 3.2.2.6)	At4g16950, At5g51630, At4g16920, At5g22690	70	70	Light and drought stress response
Disease resistance protein (TIR-NBS-LRR class) family	At5g46490, At1g56520, At1g63860, At5g45210	65	65	Salt tolerance, Osmotic stress response
Leucine-rich repeat protein kinase family protein	At5g01950, At1g51890, At1g79620, At2g27060	65	64	Growth and Disease resistance trade-off
P-loop containing nucleoside triphosphate hydrolases superfamily protein	At1g02670, At1g18410, At4g24860, At1g16800	62	63	Flowering time, Reproduction structure, UV tolerance
NAD(P)-binding Rossmann-fold superfamily protein	At5g50130, At4g23420, At2g23910, At4g00560	24	56	Shoot and root growth regulation
F-box domain-containing protein	AN1_LOCUS10777, AN1_LOCUS11011, AN1_LOCUS11578, AN1_LOCUS13042	65	54	Salt, water and cold stress response
Receptor-like serine/threonine-protein kinase (EC 2.7.11.1)	At1g61430, At4g11900, At3g16030, At1g11410	51	51	Abiotic stress response
Glycosyltransferase	AN1_LOCUS14997, AN1_LOCUS21402, AT1G05670, AT1G06000	58	46	Water and light stress response
Kinesin-like protein	At1g20060, At1g09170, At1g01950, At5g06670	43	43	Phosphate stress response
Tetratricopeptide repeat (TPR)-like superfamily protein	At1g13630, At2g33680, At5g08490, At1g16480	46	43	Light response, Metabolic and photosynthetic regulation
Xyloglucan endotransglucosylase/hydrolase (EC 2.4.1.207)	At2g14620, At3g48580, AN1_LOCUS13972	38	38	Light stress response and drought stress response
Protein DETOXIFICATION (Multidrug and toxic compound extrusion protein)	At1g61890, At4g25640, AN1_LOCUS11639, AN1_LOCUS1645,	40	39	Cold temperature stress response
2-oxoglutarate (2OG) and Fe(II)-dependent oxygenase superfamily protein	At3g19010, At2g30840, At3g49630, At4g16330	42	30	Heat, salt and drought stress responses

**Table 3:** Information for collection sites and seed sources used. Name shows the name of the location used in the manuscript and figures. Dataset column describes which dataset the location was used in. Region describes the state or area that the collection location fit into for further analysis and visualization. Elevation is listed to show the breadth of the elevational gradient ponderosa pine can exist at.

Name	Dataset	Region	Latitude	Longitude	Elevation (m)
P5S CP	One	Colorado	39.2908	−108.4915	1962.13
UT	One	Utah	37.505	−112.615	2481.072
AZ Cherry	One	Arizona	34.347	−112.04126	1610
CO WRFO83	One	Colorado	39.5832	−108.1721	2492.776611
AZ FUTS	One	Arizona	35.09543	−111.41148	2105
CO UP	One	Colorado	38.743	−108.55	2180.038574
SD BHNF	One	South Dakota	43.5655	−103.2311	1408.176
NM SFE	One	New Mexico	35.4033	−105.5381	2300
P4 CP	One	Colorado	39.296	−108.502	1989.597168
ID	One	Idaho	45.55917	−115.5528	1200
P1S CP	One	Colorado	39.2945	−108.52	2045.493896
CA SN North	One	Sierra Nevada	39.9284	−120.92667	1286.534912
CA SN South	One	Sierra Nevada	37.28705	−119.40242	942.8800049
CA HR	One	California	35.6916	−121.09126	887.1439819
WA MV	One	Washington	45.9424	−121.051	671.3024902
OR OCH	One	Oregon	44.3606	−120.457	1279.685181
WA	One	Washington	47.0448	−122.5228	106.5150909
CA SN Middle	One	Sierra Nevada	38.4748	−120.4166	1110.496704
CO STR	One	Colorado	38.249	−108.644	1755.537964
NM Sandia	One	New Mexico	35.102	−106.2264	2230
OR Des	One	Oregon	43.9529	−121.248	1273.491699
P3 CP	One	Colorado	39.28815	−108.5112	2113.887207
CO KK	One	Colorado	38.963	−108.2265	1856.239746
AZ SNBL	One	Arizona	35.201	−111.4303	2740
NE NF	One	Nebraska	42.418	−103.02	1196.34
SD BHEF	One	South Dakota	44.105	−103.373	1664.208
Doran Creek	Two	ID + WA	45.55	−114	1828.8
Hangman’s Creek	Two	MT + WY	45.48	−109.41	1371.6
CA 301	Two	CA	41.703796	−123.5694756	1371.6
Haden Creek	Two	ID + WA	48.27	−119.07	1127.76
UER	Two	AZ + NM	33.555	−109.193	1737.36
SFK Taylor CR	Two	MT + WY	45.15	−106.02	1127.76
CA 321	Two	CA	41.8997048	−122.8637599	1219.2
CA 524	Two	CA	39.9595353	−121.6057832	1066.8
Wheeler Creek	Two	ID + WA	45.5	−114.17	1828.8
Northport	Two	ID + WA	48.56	−117.48	396.24
Crystal Springs	Two	MT + WY	44.5	−107.19	1828.8
Green’s Peak	Two	AZ + NM	34.126	−109.535	2804.16
Rock Creek	Two	MT + WY	46. 52	−109.31	1463.04
Cap Gap	Two	AZ + NM	33.37	−105.28	2179.32
CA 752	Two	CA	41.1007	−120.19553	2286
Sheridan Wyoming	Two	MT + WY	44.36	−107.06	2133.6
Dominion	Two	ID + WA	48.34	−117.47	1219.2
CA 993	Two	CA	34.260498	−117.6389302	1828.8
CA 533	Two	CA	37.1262573	−119.2801494	1676.4
Mount Taylor	Two	AZ + NM	35.06	−108.08	2438.4
Cherry Road	Two	AZ + NM	34.586	−112.057	1591.9704
San Fran Peaks	Two	AZ + NM	35.33	−111.71	2743.2
Tonata Creek	Two	ID + WA	48.54	−118 44	610
Woodruff Park	Two	MT + WY	46.23	−105.36	944.88

## Data Availability

Raw sequence reads for all samples used in this study are available in the NCBI SRA under the BioProject PRJNA1241888, https://www.ncbi.nlm.nih.gov/bioproject/PRJNA1241888 (accessed on 21 October 2026). The original contributions presented in this study are included in the article. Further inquiries can be directed to the corresponding author.

## Supplementary Materials

The following supporting information can be downloaded at: https://www.mdpi.com/article/10.3390/plants15213280/s1, Figure S1. (a) PCA of seed sources in dataset two colored by genetic cluster as in Figure 1. (b) PCA putatively neutral SNPs based on HWE of seed sources in dataset two colored by region as in Figure 1. Figure S2. Pairwise Fst among seed sources shown in a neighbor joining tree for dataset two. Increased distance across the tree indicates higher pairwise Fst values and vice versa. Figure S3. IBD and IBE scatterplots. Seed Source comparisons are shown for dataset two, due to overlapping climate and geographic coordinates. Each dot represents an observed genetic distance given a specific geographic or environmental distance value. Geographic and environmental distances are shown as Euclidean distances. Genetic distance is shown as Nei’s genetic distance. Figure S4. Two-dimensional Site Frequency Spectra of genetic clusters based on all count classes for dataset two. For each axis, 1 would indicate a singleton class, 2 indicates a doubleton class, 3 tripleton class etc. X, Y points then indicate how many occurrences there are of an allele existing within a specific cluster given the specific allele count class that allele exists at in another cluster. A perfect linear line would indicate that every allele has the exact same abundance in each cluster, the further the departure from the perfect linear line indicates increasing differentiation in allele occurrence between clusters. Darker red indicates a lower number of occurrences. Darker purple indicates a higher number of occurrences. (a) 2D SFS for interior and California clusters in dataset two. (b) 2D SFS for interior and northern coastal clusters in dataset two. (c) 2D SFS for northern interior and California clusters in dataset two. (d) 2D SFS for northern interior and northern coastal clusters in dataset two. (e) 2D SFS for northern interior and interior clusters in dataset two. (f) 2D SFS for California and northern coastal clusters in dataset two. Figure S5. Interior genetic cluster full detailed demographic history. Red lines indicate mean estimates, while gray lines indicate 90,75,25,10 percent quantiles respectively. Figure S6. Northern interior genetic cluster full detailed demographic history. Red lines indicate mean estimates, while gray lines indicate 90,75,25,10 percent quantiles respectively. Figure S7. California genetic cluster full detailed demographic history. Red lines indicate mean estimates, while gray lines indicate 90,75,25,10 percent quantiles respectively. Figure S8. Northern coastal cluster full detailed demographic history. Red lines indicate mean estimates, while gray lines indicate 90,75,25,10 percent quantiles respectively. Figure S9. Climate variable importance for dataset one (a) and dataset two (b) based on gradient forest important metric. The larger the bars the larger the positive impact on prediction the variable has. Figure S10. Principal components of current G by E Space projected onto the whole range after training on 94 sampling points from dataset one (a) and 404 points (b) from dataset two. RGB values were gained from PC1, PC2, PC3 values respectively and converted to an 8-bit format. The more similar the color of a pixel the more similar the projected G by E space is and vice versa. Table S1. Pairwise FST matrix for dataset two. Wright’s Pairwise Fst was used to calculate pairwise differences. Seed source names are shown in the first row and column then pairwise Fst distances are shown in corresponding matrix cells. Table S2. K-means clustering results for dataset two. Bayesian information criterion (BIC) is used as the criterion values. Table S3. Percentage of SNPs above or below Tajima D critical values based on simulations. Purifying selection type indicates SNPs that occurred in windows below the lowest critical value. Neutral selection type indicates SNPs that occurred in windows within the upper and lower critical value. Balancing selection type indicates SNPs that occurred in windows above the highest critical value. Table S4. Coordinates and Climate Data used for gradient forest and GEA analyses. Climate data is from the 1961–1990 averages. ID represents the unique sample ID, Site represents the collection location. Dataset shows which dataset these samples belong to. Region shows State level location, Lat shows latitude, long shows longitude, Elevation shows Elevation in Meters. Table S5. Coordinates and Climate Data used for gradient forest and GEA analyses. Climate data is from the 1961–1990 averages. ID represents the unique sample ID, Site represents the collection location. Dataset shows which dataset these samples belong to. Region shows State level location, Lat shows latitude, long shows longitude, Elevation shows Elevation in Meters. Table S6. Eggnog ortholog results for GEA candidate loci in both datasets. Explanations for each column are shown in the excel file.
