# Supplementary material for: Population Structure, Demographic History and Maladaptation Risk in Ponderosa Pine
Source: Plants (Basel). 2026 Oct 24;15(21):3280. doi: 10.3390/plants15213280 (PMC13765626; doi:10.3390/plants15213280)
Supplement: Supplementary file 1 [file plants-15-03280-s001.zip › Supplementary Figures.pdf]

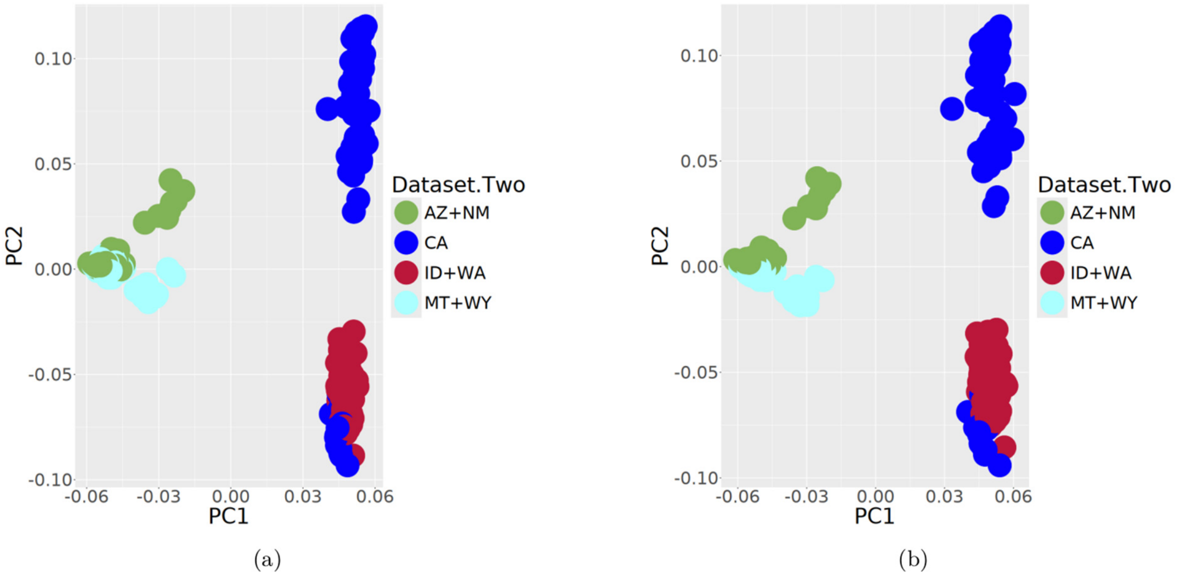

Figure S1. (a) PCA of seed sources in dataset two colored by genetic cluster as in Figure 1. (b) PCA putatively neutral SNPs based on HWE of seed sources in dataset two colored by region as in Figure 1.

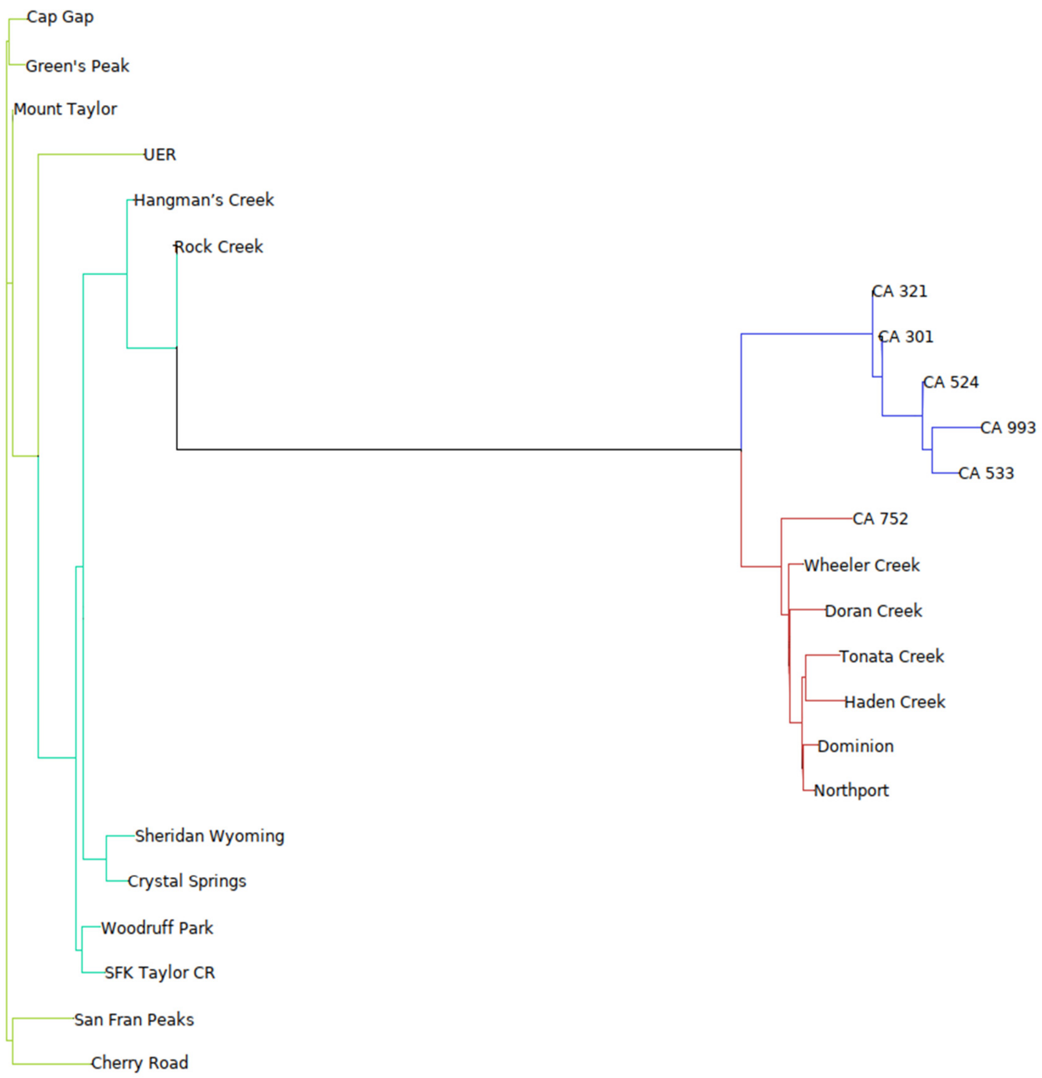

Figure S2. Pairwise Fst among sampling locations shown in a neighbor joining tree for dataset two; colored by genetic cluster from Figure 1. Increased distance across the tree indicates higher pairwise Fst values and vice versa.

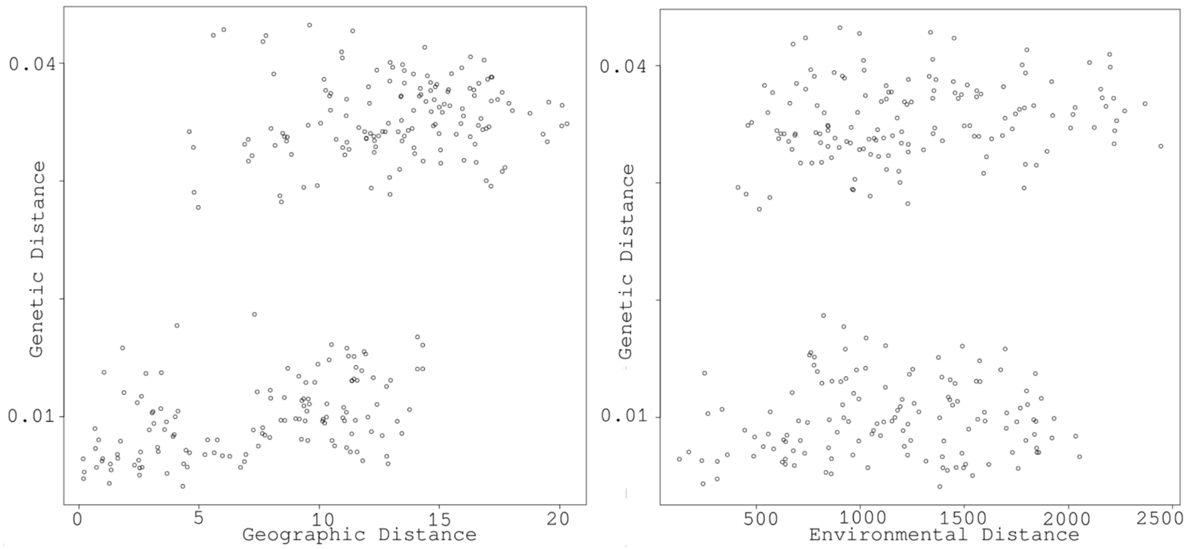

Figure S3. IBD and IBE scatterplots. Seed Source comparisons are shown for dataset two, due to overlapping climate and geographic coordinates. Each dot represents an observed genetic distance given a specific geographic or environmental distance value. Geographic and environmental distances are shown as Euclidean distances. Genetic distance is shown as Nei's genetic distance.

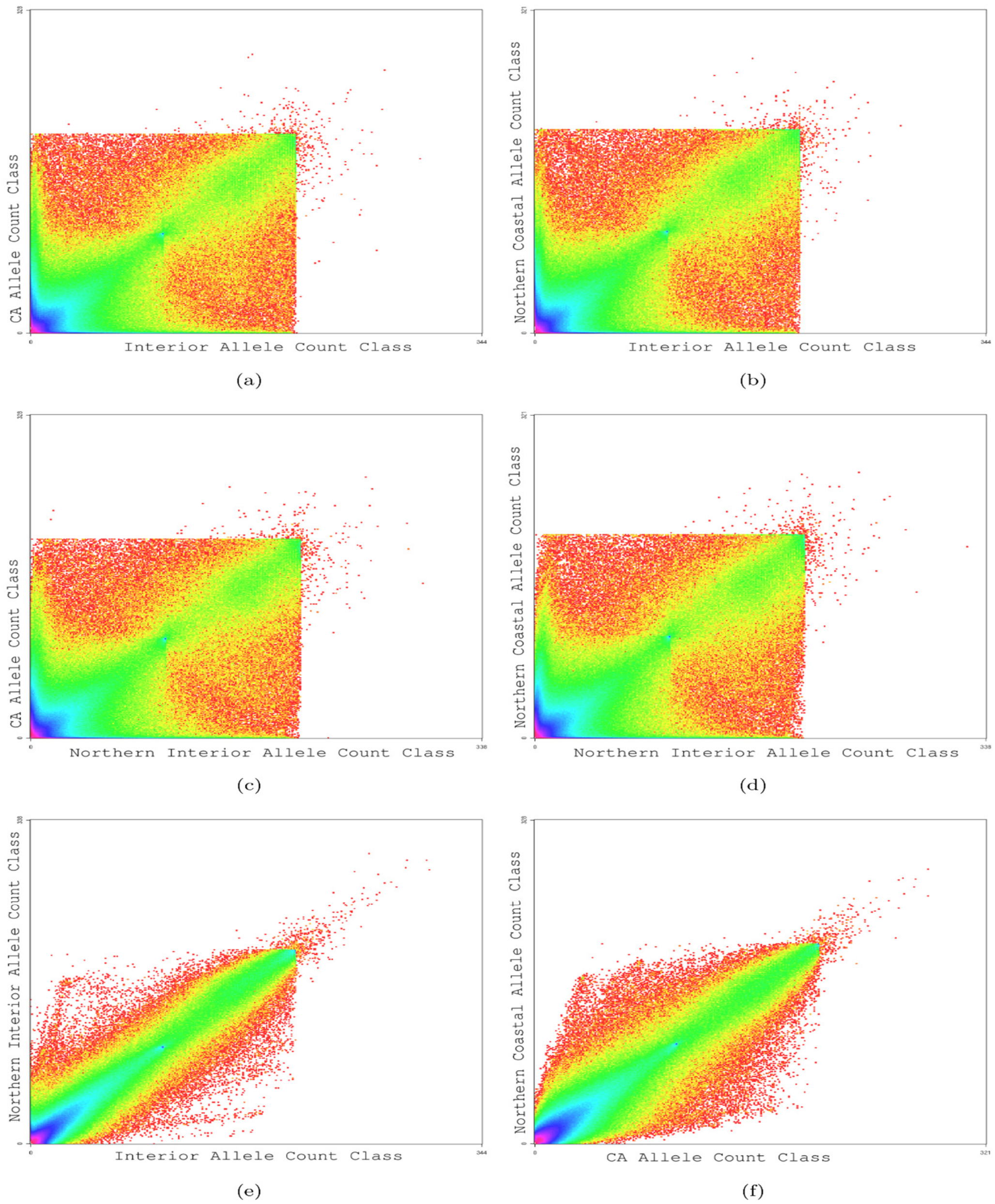

Figure S4. Two-dimensional Site Frequency Spectra of genetic clusters based on all count classes for dataset two. For each axis, 1 indicates a singleton class, 2 indicates a doubleton class, 3 tripleton class etc. X,Y points then indicate how many occurrences there are of an allele existing within a specific cluster given the specific allele count class that allele exists at in another cluster. A perfect linear line would indicate that every allele has the exact same abundance in each cluster, the further the departure from the perfect linear line indicates increasing differentiation in allele occurrence between clusters. Darker red indicates a lower number of occurrences. Darker purple indicates a higher number of occurrences. (a) 2D SFS for interior and California clusters in dataset two. (b) 2D SFS for interior and northern coastal clusters in dataset two. (c) 2D SFS for northern interior and California clusters in dataset two. (d) 2D SFS for northern interior and northern coastal clusters in dataset two. (e) 2D SFS for northern interior and interior clusters in dataset two. (f) 2D SFS for California and northern coastal clusters in dataset two.

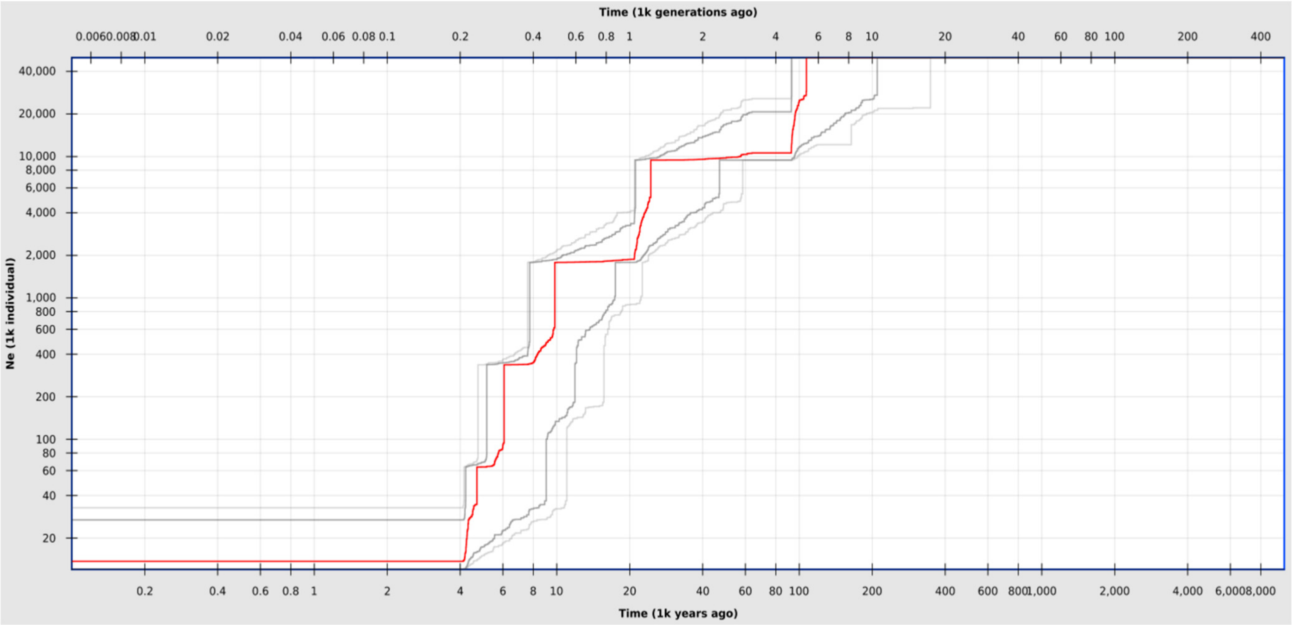

Figure S5. Interior genetic cluster full detailed demographic history. Red lines indicate mean estimates, while gray lines indicate 90,75,25,10 percent quantiles respectively

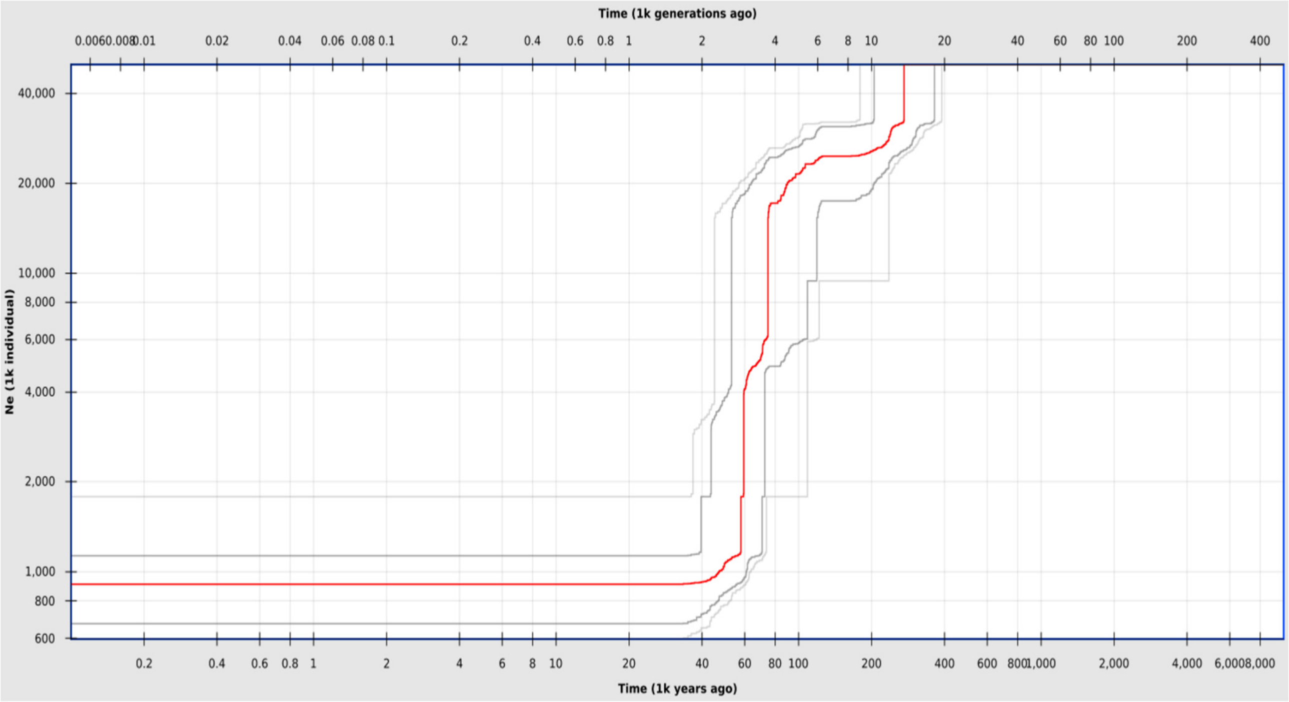

Figure S6. Northern interior genetic cluster full detailed demographic history. Red lines indicate mean estimates, while gray lines indicate 90,75,25,10 percent quantiles respectively.

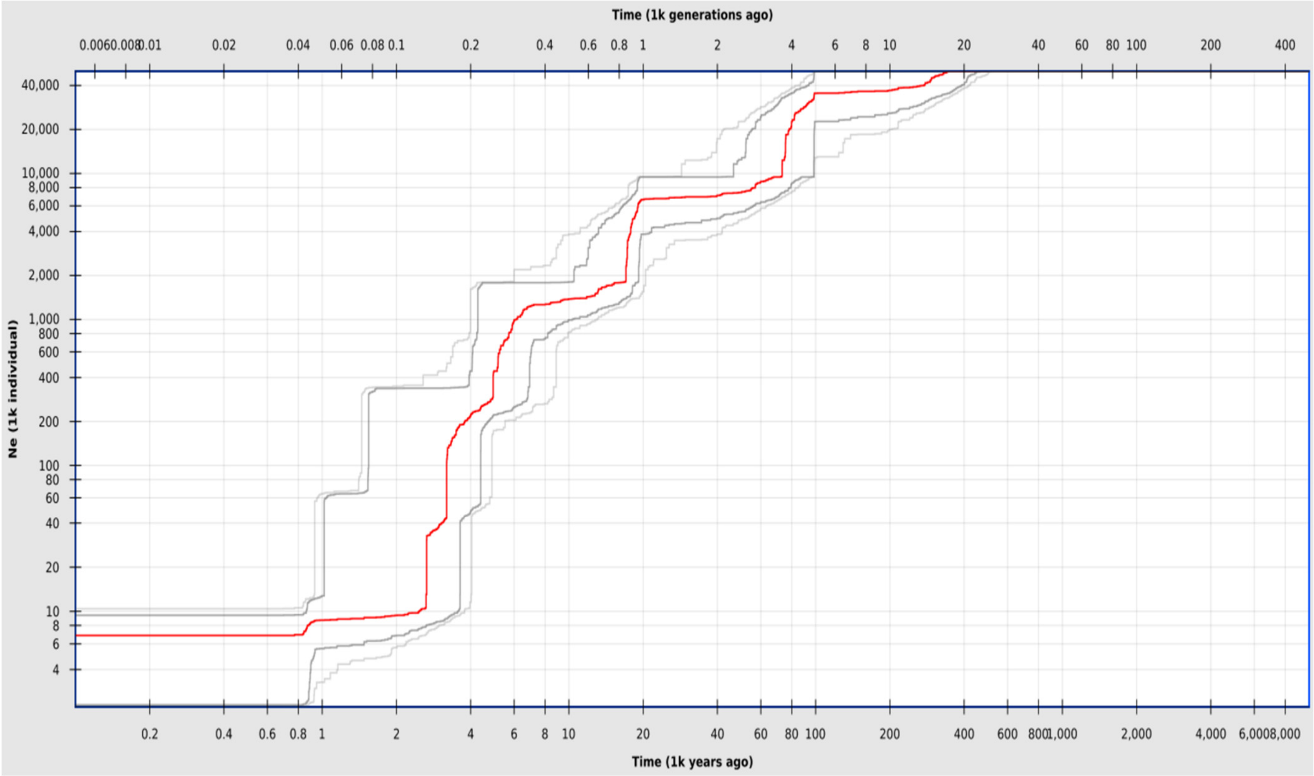

Figure S7. California genetic cluster full detailed demographic history. Red lines indicate mean estimates, while gray lines indicate 90,75,25,10 percent quantiles respectively.

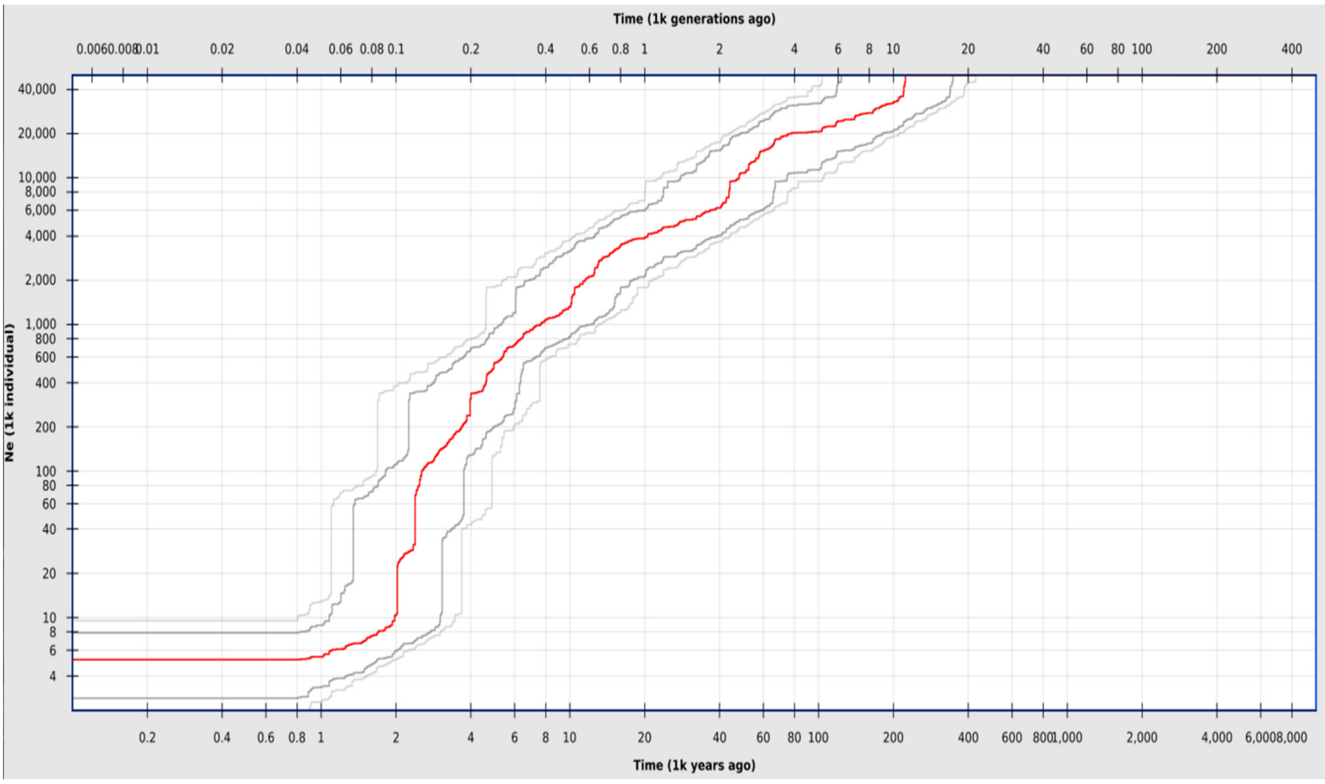

Figure S8. Northern coastal cluster full detailed demographic history. Red lines indicate mean estimates, while gray lines indicate 90,75,25,10 percent quantiles respectively.

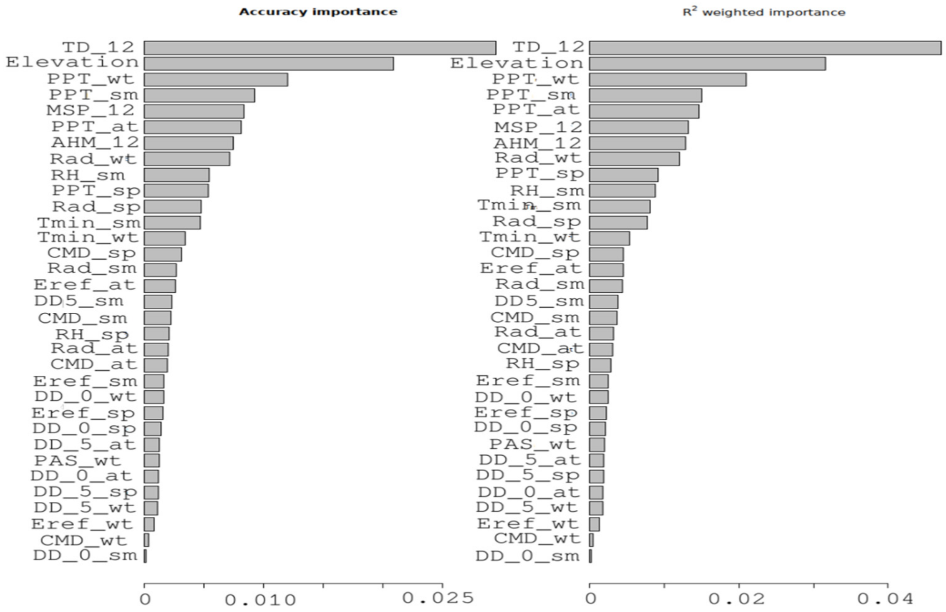

(a)

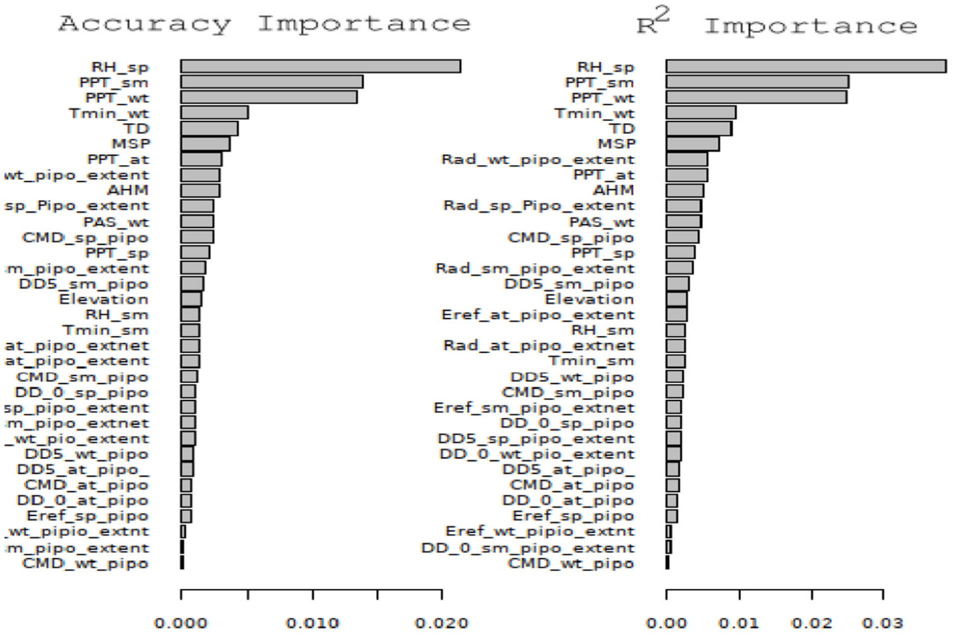

(b)

Figure S9. Climate variable importance for dataset one (a) and dataset two (b) based on gradient forest important metric. The larger the bars, the larger the positive impact on prediction the variable has.

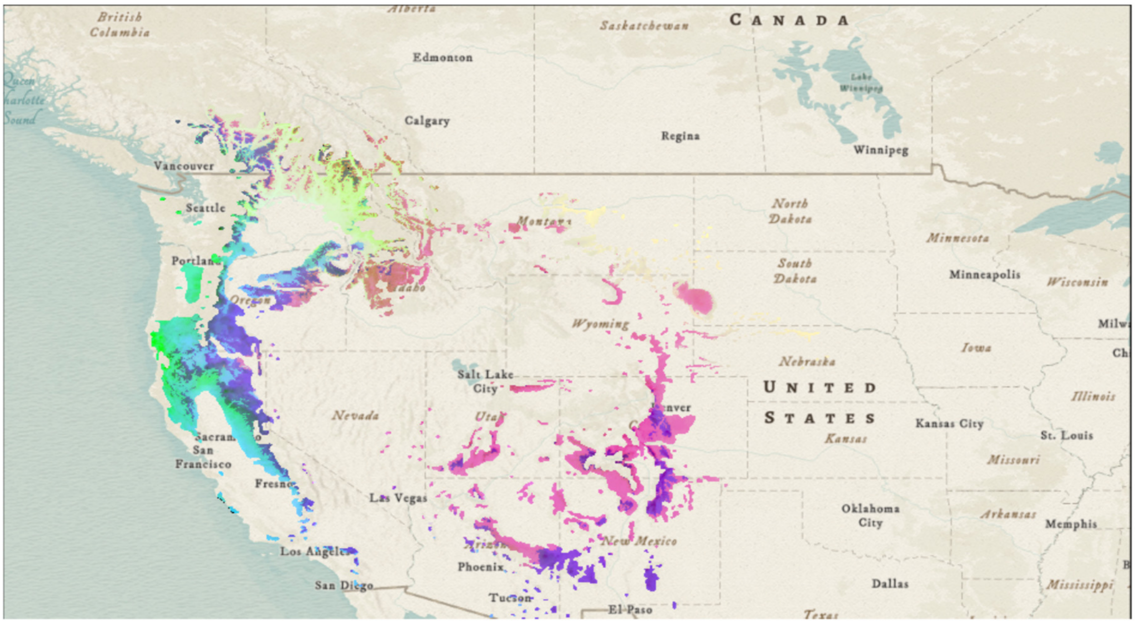

(a)

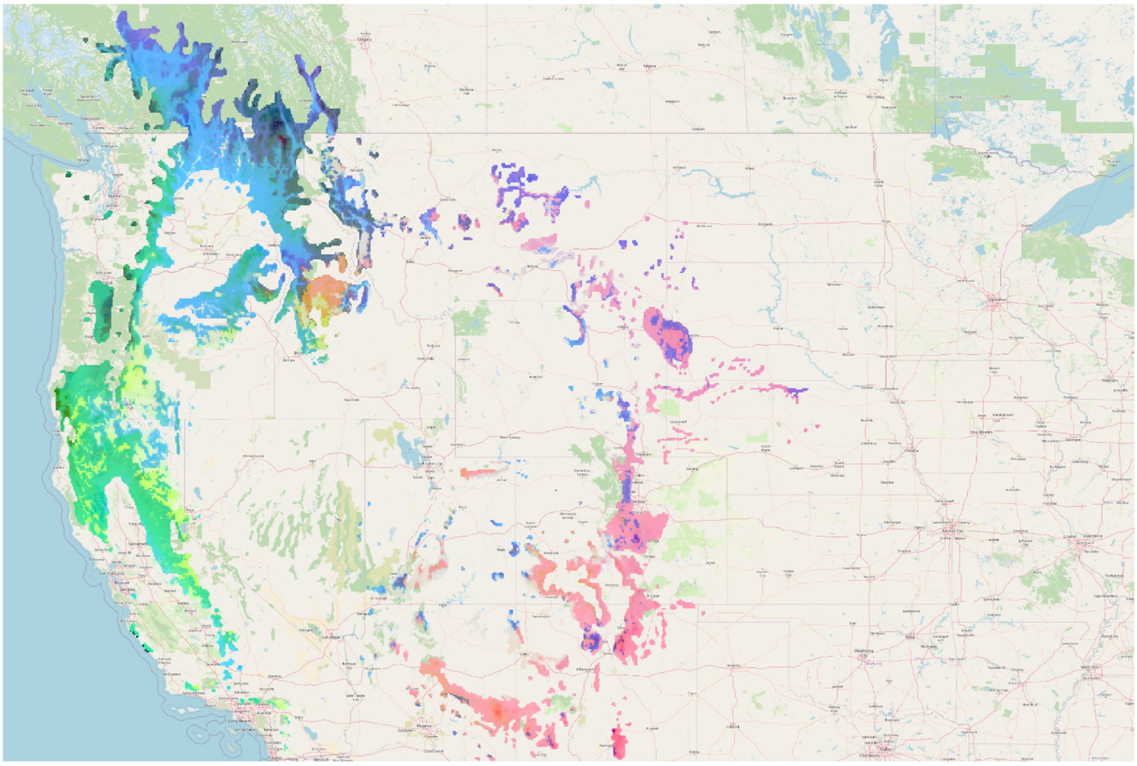

(b)

Figure S10. Principal components of current G by E Space projected onto the whole range after training on 94 sampling points from dataset one (a) and 404 points (b) from dataset two. RGB values were gained from PC1, PC2, PC3 values respectively and converted to an 8-bit format. The more similar the color of a pixel the more similar the projected G by E space is and vice versa.
